# Upper-gastrointestinal tract metabolite profile regulates glycaemic and satiety responses to meals with contrasting structure: a pilot study

**DOI:** 10.1038/s42255-025-01309-7

**Published:** 2025-06-20

**Authors:** Mingzhu Cai, Shilpa Tejpal, Martina Tashkova, Peter Ryden, Natalia Perez-Moral, Shikha Saha, Isabel Garcia-Perez, Jose Ivan Serrano Contreras, Julien Wist, Elaine Holmes, Andres Bernal, Bowen Dou, Georgia Franco Becker, Gary Frost, Cathrina Edwards

**Affiliations:** 1 Nutrition Research Section, Faculty of Medicine, Imperial College Hammersmith Campus, London, UK; 2 Food Innovation and Health, Quadram Institute Bioscience, Norwich, UK; 3 Centre for Computational and Systems Medicine, Health Futures Institute, Murdoch University, 5 Robin Warren Drive, Perth, Western Australia Australia; 4 Division of Digestive Diseases, Department of Metabolism, Digestion and Reproduction, Faculty of Medicine, Imperial College London, London, UK; 5 Chemistry Department, Universidad del Valle, Cali, Colombia

**Keywords:** Metabolomics, Duodenum, Metabolism

## Abstract

Dietary interventions to combat non-communicable diseases focus on optimizing food intake but overlook the influence of food structure. Here, we investigate how food structure influences digestion. In a randomized crossover study, ten healthy participants were fitted with nasoenteric tubes that allow simultaneous gastric and duodenal sampling, before consuming iso-nutrient chickpea meals with contrasting cellular structures. The primary outcome is gut hormone response. Secondary outcomes are intestinal content analysis, blood glucose and insulin response, subjective appetite changes and ad libitum energy intake. We show that the ‘Broken’ and ‘Intact’ cell structures of meals result in different digestive and metabolomic profiles, leading to distinct postprandial gut hormones, glycaemia and satiety responses. ‘Broken’ meal structure elicits higher glucose-dependent insulinotropic peptide, glucagon-like peptide-1 and blood glycaemia, driven by high starch digestibility and a sharp rise in gastric maltose within 30 min. ‘Intact’ meal structure produces a prolonged release of glucagon-like peptide-1 and peptide-YY, elevated duodenal amino acids and undigested starch at 120 min. This work highlights how food structure alters upper gastrointestinal nutrient-sensing hormones, providing insights into the adverse effects of modern diets on obesity and type 2 diabetes. ISRCTN registration: ISRCTN18097249.

## Abstract (web summary)

In this randomized crossover pilot study, the authors collected human gastrointestinal digesta using a unique intubation technique to compare how meals with contrasting cellular structures affect postprandial luminal metabolites, glycaemia, gut hormones and satiety sensation.

## Main

Legumes are an important crop globally with a good nutritional and environmental profile^1^. The consumption of legumes is encouraged in global non-communicable disease policies. For example, legumes have been demonstrated to improve glycaemia and affect gut hormones to prolong satiety^2,3^. However, the intake of legumes is declining globally. While processing of seeds and grains can improve palatability, this often results in the loss of plant cellular structure. Current diet and food reformulation strategies focus on reducing carbohydrate and fat intakes, but one key aspect that has been largely overlooked so far is the role of food structure in regulating the extent to which these macronutrients are released into the intestinal lumen and absorbed by the body, which has an impact on postprandial metabolism, gut hormone release and caloric value^4–6^. In raw edible plant tissues, nutrients are encapsulated within plant cells, where the surrounding plant cell wall (dietary fibre) acts as a ‘physical barrier’, protecting intracellular macronutrients from intestinal digestive enzymes and thereby limiting the release (‘bioaccessibility’) of macronutrient digestion products into the intestinal lumen^7^. This so-called ‘barrier mechanism’ is particularly well documented in cooked pulses (including chickpeas, peas and beans)^8–11^ where in vitro digestibility studies show significantly higher starch digestion from Broken cells compared with intact cells from the same source^12–15^. As the availability of glucose from starch digestion is known to be a key contributor to postprandial blood glucose response^16–18^, it has been suggested that the processing-induced breakage of chickpea cells, for example during dry-milling into flour, compromises the low glycaemic benefits that are associated with whole pulse consumption^19^. However, the proposed direct relationship between plant cell intactness, intestinal contents and the size of the glycaemic response evoked still requires confirmation^2^.

What is less well understood is how food processing-induced changes in intestinal contents impact satiety and gut hormone signalling. Of note, very recent human studies reported that subjective satiety was higher for meals containing intact chickpea cells than disrupted cells^20^, while another study showed a dose-dependent increase in anorexigenic gut hormone responses to bread incorporating Intact chickpea cells^21^.

The gut hormones such as glucose-dependent insulinotropic peptide (GIP), glucagon-like peptide-1 (GLP-1) and peptide-YY (PYY) are incretins (blood glucose-lowering effects) with established satiety-promoting effects and are produced by enteroendocrine cells in the gastrointestinal tract. GIP is produced mainly by K cells in the stomach and duodenum, whereas GLP-1 and PYY are mainly produced by L cells present in high density in the ileum and colon^22,23^. These cells have lumen-facing receptors capable of detecting and responding to local changes in intestinal nutrient concentrations. Mapping of enteroendocrine cells and receptors across the intestine tract has been well described^24,25^. A critical knowledge gap is the limited understanding of the metabolite concentrations throughout the human gastrointestinal tract, and how these change with time during meal digestion and transit. Previous work has highlighted how nutrient-sensing in the ileum stimulates GLP-1 and PYY release and promotes satiety^26–28^. However, blood GLP-1 and PYY concentrations rise within 15 min following meals^29–31^, during the period when foods are still likely to be in the stomach and duodenum.

**Fig. 1:**
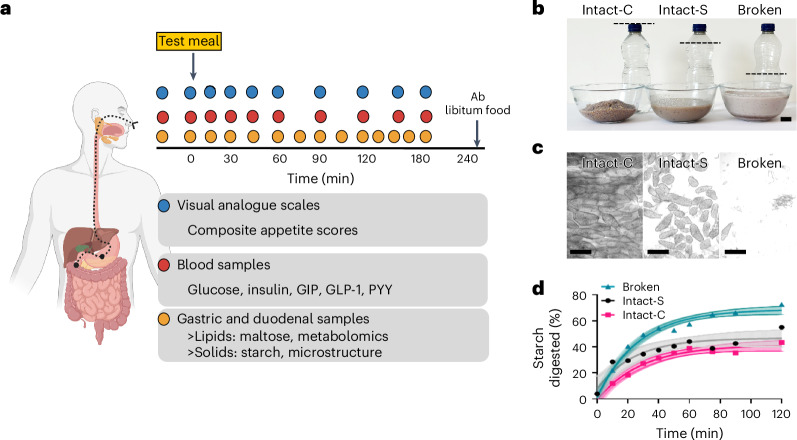
Overview of study design and test meals. **a**, Overview of study interventions. Study interventions include serial blood and intestinal sampling, VAS assessments and ad libitum food intake assessment. Image created in BioRender. **b**, Photographs of nutrient-matched test meals with contrasting structures. Flavoured cooked chickpea porridge Intact-C, Intact-S and Broken, served with water. All meal servings have the same mass and nutrient content; the different volume in the bowls reflect differences in water holding capacity. Meals consumed by human participants were freshly prepared, but these photographs are of frozen and thawed meals to enable side-by-side comparison. Dashed line shows water level. Scale bar, 20 mm (bottom right). **c**, Structural microscopy of cooked chickpea porridge. Light micrographs of cooked porridge showing clusters of Intact cells (‘Intact-C’), separate Intact cells (Intact-S) and debris from Broken cells (‘Broken’). Scale bar, 200 µm. **d**, Starch amylolysis curves show starch digestion progress during in vitro digestion with pancreatic α-amylase. Data points are means (*n* *=* 3), fitted with non-linear regression presented with s.e.m.

We combine enteric intubation techniques^32^, allowing time-resolved intestinal sampling, with parallel blood sampling to investigate the relationship between the digestive behaviour of foods with contrasting plant cell structures and the postprandial metabolic effects. This study investigated the effect of chickpea cotyledon cell intactness, as a model of legume processing, on digestion product concentrations within the gastric and duodenal lumen and its consequences for postprandial blood glucose, insulin and gut hormone responses. Chickpea seeds were cooked and processed differentially to achieve three contrasting meal structures: ‘Intact-C’ were tissue macroparticles (intact cell clusters); ‘Intact-S’ were separated intact cells; and ‘Broken’ were blended before cooking to break open every cell. (Fig. 1c; structures confirmed by light microscopy). This work investigated human gastric and duodenal metabolites using ^1^H-nuclear magnetic resonance (NMR) profiling^33^ to explore the luminal content regulating appetite-regulating hormone responses. Despite their identical macronutrient and fibre content, the non-cellular chickpea meals were hypothesized to be more rapidly digested and thereby cause higher glycaemic responses^19,34^ than the cellular chickpea meals. Furthermore, the Intact-C and Intact-S meals were hypothesized to retain their structure within the stomach and duodenum, resulting in different digestibility and thereby luminal metabolic profiles capable of prolonging gut hormone and satiety responses.

## Results

### Participants and diets

**Extended Data Fig. 1:**
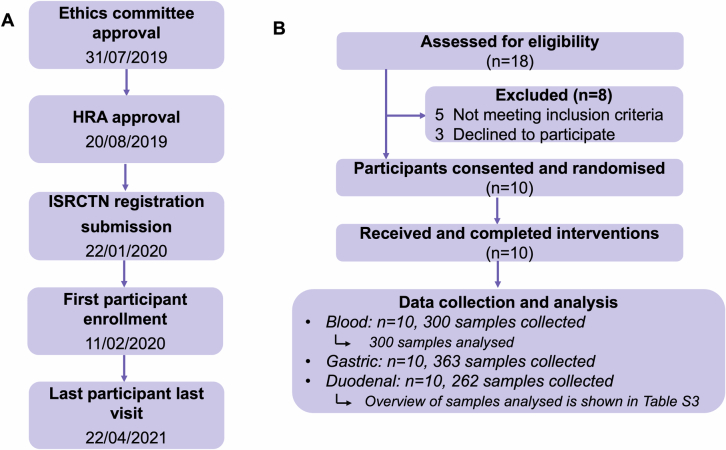
Study flowchart. (**a**) Study key dates and (**b**) CONSORT flow diagram illustrating the study procedures, data collection and analysis. Missing data was described in Supplementary Table 3. HRA, Health Research Authority.

We admitted six male and four female adult participants aged (mean ± s.e.m.) 30.8 ± 2.4 years with body mass index (BMI) 24.9 ± 0.8 kg m^−2^ and fasted glucose 4.7 ± 0.1 mmol l^−1^ (Supplementary Tables 1 and 2 provide eligibility criteria and participant characteristics) as inpatients to the National Institute for Health and Care Research (NIHR) Imperial Clinical Research Facility (CRF) at Hammersmith Hospital in London, UK, where they resided for 4 days. The sex of participants was determined based on self-report and presentation and was not used in the selection of study participants. On day 1, they were fitted with two enteral feeding tubes to enable concomitant sampling from the participants’ stomach and upper small intestine (duodenum). On the morning of days 2, 3 and 4, participants received one of three structurally different test meals in random order, and samples of blood, intestinal content and appetite visual analogue scale (VAS) scores were collected up to 3 h postprandially as shown in Fig. 1a. A consort diagram showing flow of participants through the study is provided in Extended Data Fig. 1.

The test meals (Fig. 1b–d) consisted of freshly cooked chickpea porridge in water, flavoured with low-sugar blackcurrant jam and raspberry flavoured jelly (shown in photographs in Fig. 1b). All meals had the same nutrient content per serving (~242.7 kcal, 29.5 g starch, 2.3 g sugar, 6.6 g dietary fibre, 11.0 g protein and 3.7 g fat) but had been processed differently (Supplementary Text 1) so that these nutrients were either entrapped within clusters of cells ‘Intact-C’, isolated single cells ‘Intact-S’ or broken cells ‘Broken’, as confirmed with light microscopy (Fig. 1c). Using an in vitro starch amylolysis assay, we showed that these differences in meal structure affected starch susceptibility to α-amylase digestion, with the Broken cell meal showing the greatest release of starch digestion products (Fig. 1d).

### Non-cellular structure elevated blood glucose and insulin

**Fig. 2:**
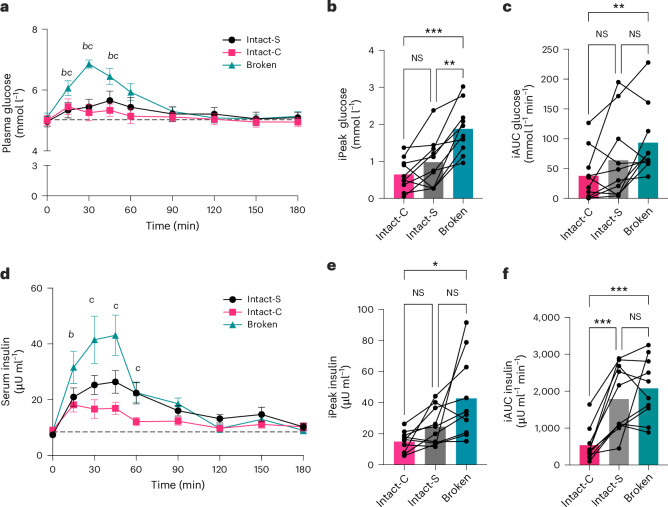
Blood glucose and insulin responses to chickpea meals with contrasting structures. **a**–**f**, Postprandial (3 h) blood glucose (**a**–**c**) and insulin (**d**–**f**) responses in healthy participants (*n* *=* 10) to macronutrient-matched chickpea meals (30 g starch per serving) in which cotyledon cells are present as cell clusters ‘Intact-C’, separated cells ‘Intact-S’, or no longer cellular ‘Broken’. Time-series (**a**,**d**) show mean with s.e.m. (*n* *=* 10). Two-way RM ANOVA showed significant meal × time effects for glucose (*P* *<* 0.001) and insulin (*P* *=* 0.003) and time-point annotations show significant differences (adj. *P* *<* 0.05, Tukey’s) based on post hoc pairwise comparisons. Significant differences between interventions are annotated: *b*, Intact-S vs Broken; *c*, Intact-C vs Broken. Intact-S versus Intact-C (**a**). Intact-S versus Broken (**b**). Intact-C versus Broken (**c**). Bar charts show maximum rise from fasted concentrations (‘iPeak’) and (CF) incremental AUC for glucose (**b**,**c**) and insulin (**e**,**f**), where bars show the mean responses to each meal and connected data points are from the same individual. One-way RM ANOVA showed significant main meal effects on iPeak (*P* *<* 0.001 and *P* *=* 0.015 for glucose and insulin) and iAUC (*P* *=* 0.008 and *P* *<* 0.001 for glucose and insulin) and annotations are the result of post hoc pairwise comparison; Tukey’s multiplicity adj. *P* values are *P *<* 0.05, **P *<* 0.01, ***P *<* 0.001, NS, not significant. Source data

Meal cell structure significantly influenced postprandial glucose (*P* < 0.001) and insulin (*P* = 0.003) as shown in Fig. 2a,d. The magnitude of glucose and insulin response was assessed using the incremental peak (iPeak) and incremental area under the curve (iAUC), revealing significant differences between Broken and Intact-cellular meals. Broken cells led to a 190% increase in iPeak glucose compared with Intact-C (1.23 ± 0.56 mmol l^−1^) and a 92% increase compared with Intact-S (0.90 ± 0.50 mmol l^−1^) (Fig. 2b). Similarly, insulin iPeak after ‘Broken’ meals was 65% higher than after ‘Intact-C’ (−27.76 ± 24.44 μU ml^−1^, adj. *P* = 0.028; Fig. 2d). Glucose iAUC was 148% higher after consuming Broken compared with Intact-C (55.78 ± 36.56 mmol l^−1^ min^−1^, adj. *P* = 0.005) (Fig. 2c). Broken also resulted in a 74% higher insulin iAUC (−1,538.92 ± 706.72 μU ml^−1^, adj.*P* < 0.001) compared with Intact-C.

Timing of glucose peaks occurred at approximately 34 min and did not differ between Broken and Intact meals (*P* = 0.173; Supplementary Table 4), likely suggesting their rates of gastric emptying were similar^35^. Intact-C had a shorter insulin response, with a mean duration of 76 min less to return to baseline levels compared with Broken and Intact-S (Supplementary Table 4; adj. *P* = 0.004 and 0.036). This can be attributed to the reduced demand for insulin secretion in the Intact-C group given the lower blood glucose levels.

Overall, the chickpea porridge made from Broken cells caused the highest glycaemic response, whereas cell clusters had the lowest impact on blood glucose and insulin. Chickpeas have been widely described as low glycaemic foods^36^, whereas our data clearly show that disruption of meal cell structures significantly increases the magnitude and duration of the postprandial blood glucose and insulin response.

### Intact-cellular structure enhanced GLP-1, PYY and satiety

**Fig. 3:**
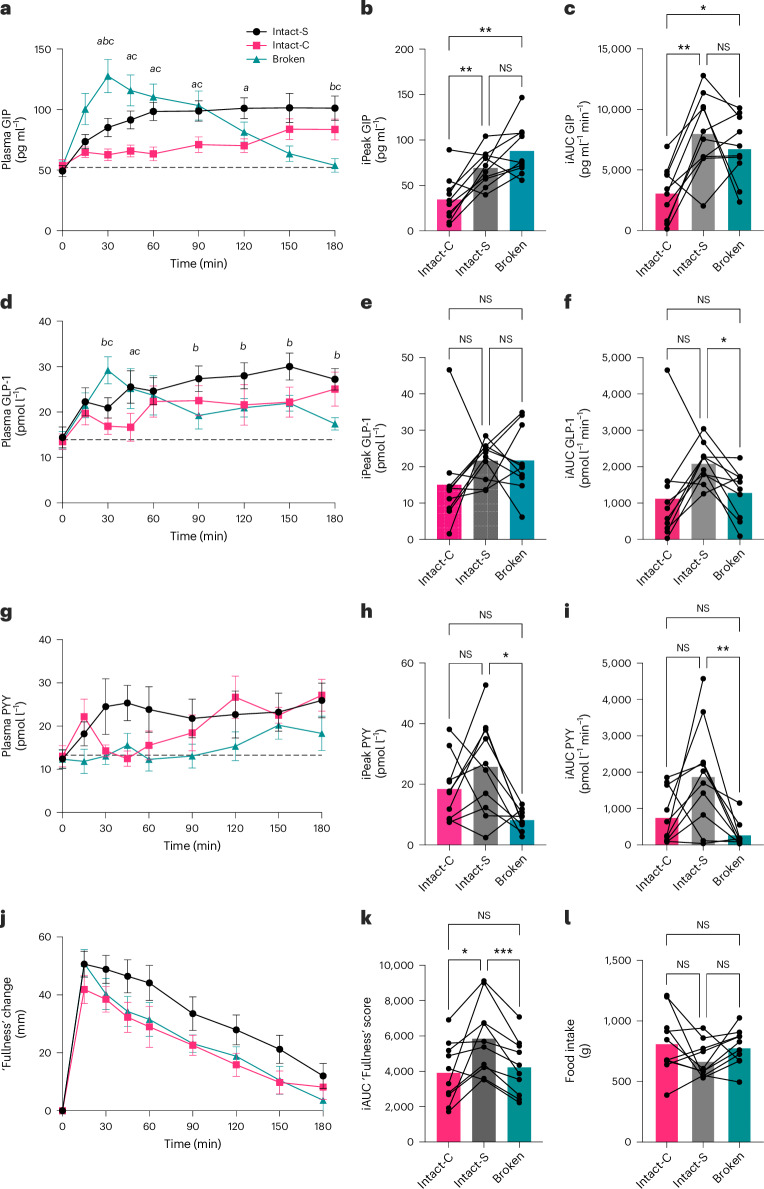
Gut hormone and appetite responses to chickpea meals with contrasting structures. **a**–**l**, Postprandial (3 h) blood GIP (**a**–**c**), GLP-1 (**d**–**f**), PYY (**g**–**i**) and subjective appetite (**j**,**k**) responses and ad libitum food intake (**l**) in healthy participants (*n* = 10) to macronutrient-matched chickpea meals (30 g starch per serving) in which cotyledon cells are present as cell clusters ‘Intact-C’, separated cells ‘Intact-S’ or no longer cellular ‘Broken’. Time-series (**a**,**d**,**g**,**j**) show mean with s.e.m. (*n* = 10). Two-way RM ANOVA showed significant meal × time effects for GIP (*P* < 0.001) and GLP-1 (*P* = 0.034) and main effects for PYY (*P* = 0.003) and time-point annotations show significant differences (adj. *P* < 0.05, Tukey’s) based on post hoc pairwise comparisons. Significant differences between interventions are annotated: *a*, Intact-S vs Intact-C; *b*, Intact-S vs Broken ; *c*, Intact-C vs Broken. Intact-S versus Intact-C (**a**). Intact-S versus Broken (**b**). Intact-C versus Broken (**c**). Bar charts show maximum rise from fasted concentrations (iPeak) and (**c**,**f**,**i**) iAUC for GIP (**b**,**c**), GLP-1 (**e**,**f**) and PYY (**h**,**i**), where bars show mean responses to each meal and data points connected by a line are from the same individual. A one-way RM ANOVA showed significant main meal effects on iPeak (*P* = 0.002 and *P* = 0.144, *P* = 0.012 for GIP, GLP-1 and PYY) and iAUC (*P* = 0.002, *P* = 0.070 and *P* = 0.008 for GIP, GLP-1 and PYY) and annotations are the result of post hoc pairwise comparisons; Tukey’s multiplicity adj. *P* values are *P < 0.05, **P < 0.01, ***P < 0.001. Bar chart (**k**,**l**) shows incremental area of fullness and ad libitum food intake (4 h) where bars show mean responses to each meal and data points connected by a line are from the same individual. A one-way RM ANOVA showed a significant main effect on fullness score (*P* = 0.007) but not on food intake (*P* = 0.176). Source data

Fasting levels of blood GIP, GLP-1 and PYY did not differ between intervention days, and the within-subject variabilities across interventions were relatively low (Supplementary Table 9). Figure 3 shows how meal structure impacted postprandial blood GIP, GLP-1 and PYY responses. There was a significant time × meal effect on postprandial GIP (*P* < 0.001), with very clear differences between meal types evident in the time-series data shown in Fig. 3a. After Intact-S and Intact-C meals, GIP concentrations increased slowly to reach a maximum after ~120 min, but following the Broken meal, GIP increased rapidly to a higher peak after ~30 min then declined gradually to reach fasted levels after ~3 h (Fig. 3a). GIP concentrations (iPeak values) for Intact-C (Fig. 3b) were 61% lower than the Broken (53.47 ± 37.84 pg ml^−1^, adj. *P* = 0.009) and 50% lower than Intact-S (34.77 ± 23.20 pg ml^−1^, adj. *P* = 0.006); the iPeak of Intact-S and Broken was comparable (adj. *P* = 0.128). Similarly, the iAUC of Intact-C (Fig. 3c) was 54% lower than Broken (3,663.32 ± 3,397.32 pg ml^−1^ min^−1^, adj. *P* = 0.036) and 61% lower than Intact-S (4,924.82 ± 2,858.02 pg ml^−1^ min^−1^, adj. *P* = 0.003). Intact-S and Broken showed no significant difference in GIP iAUC (adj. *P* = 0.559). Thus, the chickpea porridge containing Broken plant cells caused a large and early spike in blood GIP, whereas the cellular meals elicited a more gradual and sustained GIP response.

GLP-1 responses were also significantly altered by meal structure (time × meal interaction, *P* = 0.034) and followed similar time trends to GIP. Fig. 3d shows that GLP-1 increased gradually after ingestion of Intact-S and Intact-C, whereas the Broken meal caused GLP-1 concentration to reach a higher peak after ~30 min. The maximum postprandial rise was comparable in the three groups (Fig. 3e; *P* = 0.14), while the Intact-S reached the peak 84 min later compared with Broken (adj. *P* = 0.003; Supplementary Table 4). iAUC was 63% higher in Intact-S compared with Broken (Fig. 3f; 801.55 ± 661.0 pmol l^−1^ min^−1^, adj. *P* = 0.020). Thus, the Broken cell meal elicited high GLP-1 concentrations in the early-postprandial period, whereas cellular meals caused GLP-1 concentrations to reach a similar level, but later in the postprandial period (90–180 min).

Meal structure significantly affected PYY response (main effect, *P* = 0.003; Fig. 3g). Intact-S elicited a higher PYY response compared with Broken: PYY iPeak for Intact-S was 214% higher (17.53 ± 13.00 pmol l^−1^) than Broken (adj. *P* = 0.012; Fig. 3h). Intact-S prolonged PYY response, as evidenced by Intact-S approaching baselines 70 min later than Broken (adj. *P* = 0.021, Supplementary Table 4). The first peak iAUC for Intact-S was 1,604.63 ± 1,113.88 pmol l^−1^ min^−1^ (568%) higher than Broken (adj. *P* = 0.008; Fig. 3i). Overall, ingestion of chickpea porridge with Intact separated cells (Intact-S) caused PYY concentrations to increase during the first hour of meal ingestion and remain elevated, whereas ingestion of Broken cells resulted in a delayed and lower PYY response.

**Extended Data Fig. 2:**
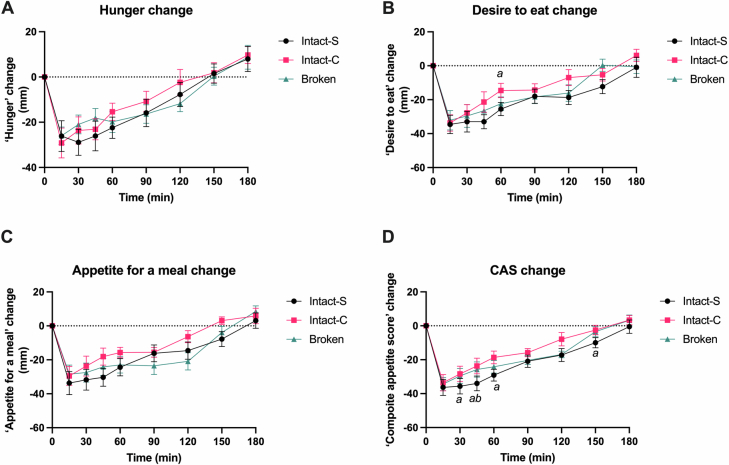
Subjective appetite scores responses to meal type. Delta changes in feeling of hunger (**a**), desire to eat (**b**), and appetite for a meal (**c**) and composite appetite score (CAS) (**D**) following consumption of chickpea test meals. Data presented as mean ± SEM (n = 10). Two-way repeated-measures ANOVA showed significant meal x time effects for desire to eat (P = 0.029) and composite appetite score (P < 0.001) but not for feeling of hunger or appetite for a meal; and time point annotations show significant differences differences (adj. P < 0.05, Tukey’s) based on two-sided post hoc pairwise comparisons: a Intact-S vs Intact-C; **b** Intact-S vs Broken; and c Intact-**C** vs Broken. Source data

PYY and GLP-1 have the potential to impact appetite, so to explore this further we captured the ten participant’s responses (VAS scores) to fullness/appetite questions. Postprandial changes in fullness significantly differed between meal structures (Fig. 3j, meal effect; *P* = 0.007); with Intact-S resulting in higher ‘fullness’ scores compared with Intact-C and Broken (Fig. 3k; adj. *P* = 0.031 and *P* < 0.001, respectively). No significant differences were observed for ‘hunger’ and ‘appetite for a meal’ (meal effect, *P* = 0.606 and 0.060, respectively), but ‘desire to eat’ showed a significant meal effect (*P* = 0.029), as did the overall composite appetite scores (meal effect, *P* < 0.001, calculated as the mean of ‘hunger’, ‘appetite for a meal’, ‘desire to eat’ and (100 – ‘fullness’); Extended Data Fig. 2). These indicated that Intact-S resulted in lower overall appetite compared with other meals. Ad libitum food intake measured at postprandial 240 min did not show significant differences between meals (Fig. 3l, meal effect; *P* = 0.123).

The finding that Intact-C had a lower GIP response than Intact-S, could be explained by the lower glucose release during earlier stages of digestion from Intact-C. This is supported by Fig. 5, which shows that gastric glucose/maltose concentrations decrease at a similar rate for both meals, but tend to be higher after Intact-S than Intact-C. Assuming that these differences persist throughout the small intestine, it is plausible that maltose/glucose and other digestion products would be released later from Intact-C, which could explain the lower GLP-1 and later PYY response for Intact-C compared with Intact-S.

Our findings are consistent with another recent study that also found that consuming chickpea Intact cells led to enhanced satiety compared with Broken cells, without reducing food intake^20^. It is unclear why the participants’ higher satiety scores did not result in lower food intake at the subsequent meal. One possibility is that the relatively low energy density of the chickpea meals (for instance, ~500 ml of the ‘Broken’ meal is required to provide 242.7 kcal), and short eating duration may not have induced a sufficient response to impact food intake at 4 h. Future study designs should consider exploring cell intactness within a more energy dense meal and impacts on satiety responses and subsequent food intake.

Overall, these results showed chickpea separated cells enhanced GLP-1 and PYY responses and appetite suppression. These are consistent with a very recent study on meals containing legume cells^21^; our study uniquely controlled for micronutrient and fibre contents, demonstrating that the structure-derived effects would be sufficient to influence postprandial satiety through nutrient-mediated gut hormone signalling.

### Cell wall intactness modulates upper gut starch digestion

Gastric and duodenal aspirates were collected from ten participants and examined in terms of structure and composition to explore how differences in meal structure alter intestinal content, potentially generating signals which explain observed differences in blood responses.

**Fig. 4:**
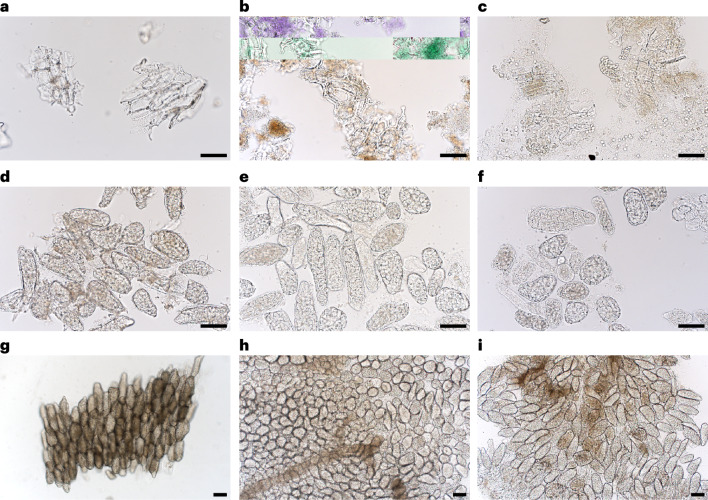
Micrographs of chickpea cell structures during gastric and duodenal digestion. **a**–**i**, Light micrographs showing characteristics structures from Broken (**a**–**c**), Intact-S (**d**–**f**) and Intact-C (**g**–**i**) meals (**a**,**d**,**g**) and digesta aspirated from the stomach (**b**,**e**,**h**) and duodenum (**c**,**f**,**i**). All aspirates from all participants were imaged a minimum of ten times at different magnifications, so in total, over 5,700 images were acquired, and the representative images shown here were selected from 61 gastric and 105 duodenal aspirates captured from the digesta of the same individual. Scale bars, 100 µm. Source data

Light microscopy was performed on aspirated stomach and duodenal content to gather insight into the structural breakdown of these meals within the human gastrointestinal lumen. Light micrographs (Fig. 4) show that the characteristic meal structures, which are non-cellular for Broken (Fig. 4a–c), cellular for Intact-S (Fig. 4d–f) and cellular clusters for Intact-C (Fig. 4e–g), persisted within the stomach and duodenum, despite oral processing. Whole cells or cell clusters were found in the duodenum 15 min after meal ingestion and were present in every sample throughout the entire 180 min of sample collection. This was consistent for all participants. For the cellular meals, there was no evidence of progressive structural breakdown of plant cells with gastrointestinal residence time or region (stomach versus duodenum). When cells were ingested as clusters, clusters were clearly visible to the naked eye within the digesta, these were successfully captured using light microscopy, although some images give the illusion of cell separation, which results from pressing on the coverslip. Thus, the clusters can break down into separated cells, although this process could not be captured quantitatively. No Intact chickpea cells were detected after ingestion of the Broken cell meals. After ingestion of Broken cell chickpea porridge, partially digested starch granules were evident already within the first collection point from the stomach.

**Fig. 5:**
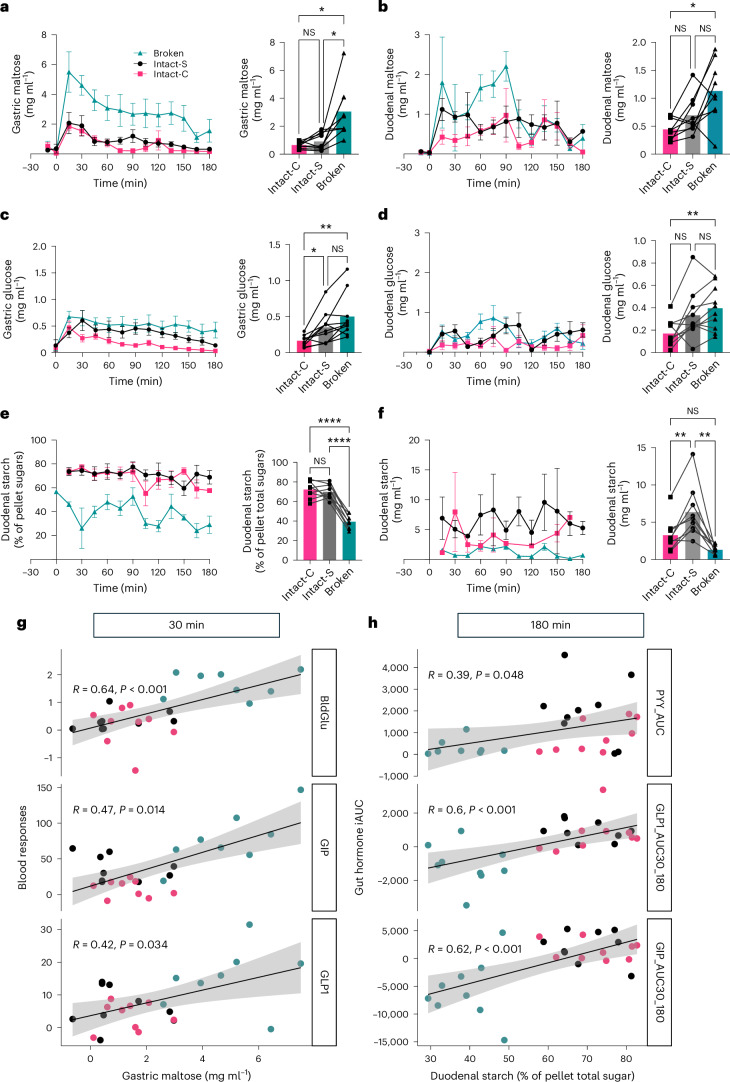
Intestinal concentrations of starch digestion products, estimated undigested starch contents and their relationship with blood responses. **a**, Time-series and average concentrations for gastric maltose. Connected data points in bar charts are from the same individual. Data show mean ± s.e.m., *n* = 9. **b**, Time-series and average concentrations for duodenal maltose. Connected data points are from the same individual. Data show mean ± s.e.m., *n* = 9. **c**, Time-series and average concentrations for gastric glucose. Connected data points are from the same individual. Data show mean ± s.e.m., *n* = 9. **d**, Time-series and average concentrations for duodenal glucose. Connected data points are from the same individual. Data show mean with s.e.m., *n* = 9. **e**, Time-series and average concentrations for duodenal undigested starch expressed as a proportion of ‘total pellet sugars’. Connected data points are from the same individual. Data show mean ± s.e.m., *n* = 9. **f**, Time-series and average concentrations for duodenal undigested starch calculated from the aspirated solid content and its pellet composition. Data shows mean with s.e.m., *n* = 9. **g**, Spearman correlations of 0–30 min delta changes in gastric maltose (mg ml^−1^) with blood glucose (mmol l^−1^), GIP (pg ml^−1^) and GLP-1 (pmol l^−1^). The scatter-plot displayed individual points with a regression line representing the mean predicted values and shaded bands representing 95% CI (*n* = 9). BldGlu, blood glucose. **h**, Spearman correlations of undigested starch in duodenal aspirates with PYY iAUC pmol l^−1^ × (0–180 min), GLP-1 iAUC pmol l^−1^ × (30–180 min) and GIP iAUC pg ml^−1^ × (30–180 min). The scatter-plot displayed individual points with a regression line representing the mean predicted values and shaded bands representing 95% CI (*n* = 9). For bar charts ABCD, one-way RM ANOVA showed significant main effects for gastric maltose (*P* = 0.006), gastric glucose (*P* = 0.002), duodenal maltose (*P* = 0.020) and duodenal glucose (*P* = 0.003) and annotations are the result of two-sided post hoc pairwise comparisons; Tukey’s multiplicity adj. *P* values were *P < 0.05, **P < 0.01, ****P < 0.001. For scatter-plot (EF), two-sided Spearman correlation tests showed that gastric maltose was significantly correlated with blood glucose (*R* = 0.64, *P* = 0.004), GIP (*R* = 0.47, *P* = 0.014) and GLP-1 (*R* = 0.42, *P* = 0.034). Duodenal undigested starch was significantly associated with PYY iAUC (0–180 min) (*R* = 0.39, *P* = 0.048), GLP-1 iAUC (30–180 min) (*R* = 0.60, *P* < 0.001) and GIP iAUC (30–180 min) (*R* = 0.62, *P* < 0.001). Source data

In summary, the microscopic analyses showed that the cellular meals largely remained intact in the gastric and duodenal aspirates during the entire postprandial sampling period, thus providing effective barriers as starch remained encapsulated within those plant cells.

The effects of meal structure on starch and starch digestion products within the aspirated fluid are shown in Fig. 5. Starch digestion products (maltose > maltotriose > glucose) were present in postprandial (0–3 h) gastric and duodenal aspirate supernatants, and gastric concentrations were higher than duodenal. Maltose (Fig. 5a,b), being the main product of starch amylolysis, was present at ~50% higher concentrations than maltotriose (Supplementary Table 5) and glucose (Fig. 5c,d). Gastric maltose concentrations (Fig. 5a) differed between meal types (*P* = 0.006, one-way repeated-measures (RM) analysis of variance (ANOVA)). Pairwise comparison (Tukey’s) showed that the Broken meal elicited a 2.13 ± 1.85 mg ml^−1^ (233%) and 2.39 ± 1.74 mg ml^−1^ (370%) higher gastric maltose response than both cellular meals Intact-S (adj. *P* = 0.027) and Intact-C (adj. *P* = 0.011), while there was no difference between Intact-S and Intact-C. Sucrose concentrations (Supplementary Table 5), included as a meal marker, were not significantly different in gastric aspirates between meal types (*P* = 0.055 for both gastric samples) and the time-series data indicated a gradual drop in gastric sucrose concentrations (presumably reflecting liquid-phase gastric emptying, suggesting that most of the digesta had been emptied from the stomach from ~60 min).

**Extended Data Fig. 3:**
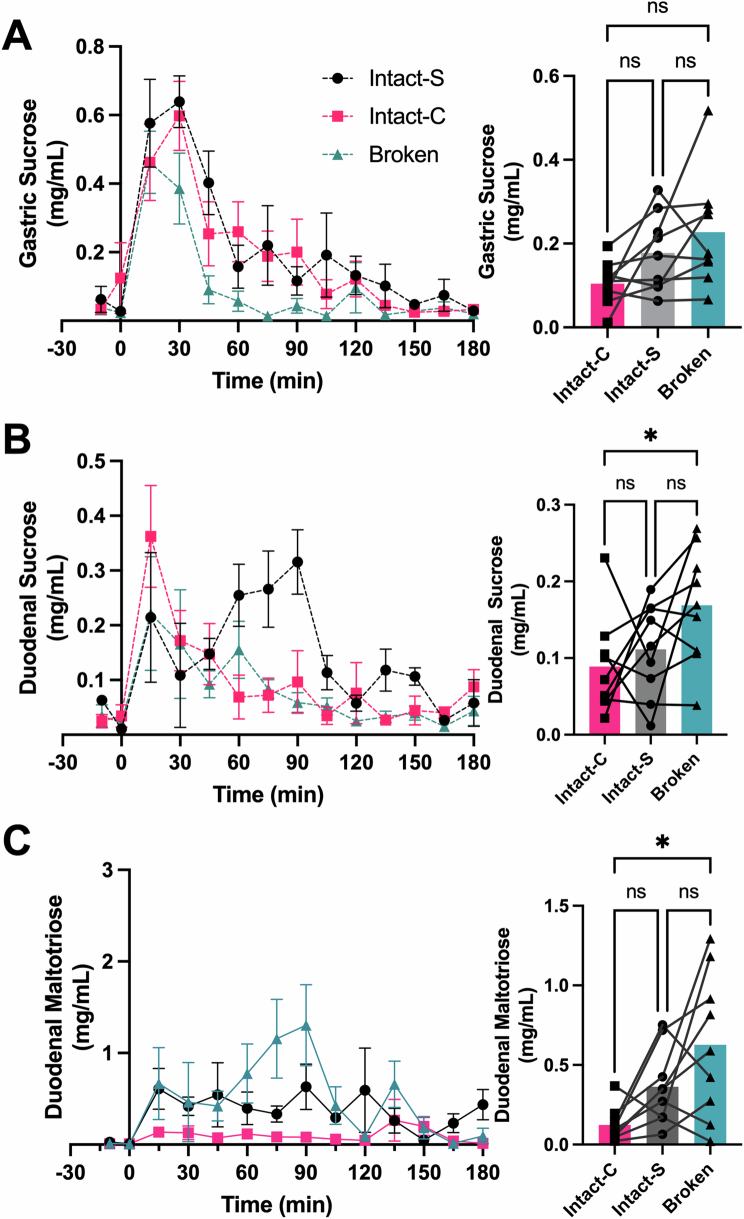
Intestinal carbohydrates responses to meal type. Intestinal concentrations of gastric sucrose (**A**), duodenal sucrose (**B**) and maltotriose (**C**) following consumption of chickpea test meals. Time-series data (mean with SEM) showed analyte concentrations measured at time-points to up 3 h after each meal structure using samples from 9 participants. Bar charts showed average concentrations of each analyte over the 3 h period and connected data points are from the same individual. Repeated-measures one-way mixed effects ANOVA was performed on each bart chart; annotations refer to multiplicity adjusted P values from pairwise comparisons using Tukey’s *post hoc* test: *p < 0.05, **p < 0.01, ***p < 0.001. One-way ANOVA showed non-significant effect on gastric sucrose (p = 0.055, bar chart **A**), significant meal effect on average 0–3 h duodenal sucrose (p = 0.041, bar chart **B**) and duodenal maltotriose (p = 0.038, bar chart **C**). Source data

Duodenal maltose concentrations (Fig. 5b) were also significantly different between meal types (*P* = 0.020*)*, with pairwise comparisons showing that the Broken meal elicited 0.689 ± 0.686 mg ml^−1^ (140%) higher duodenal maltose concentrations than Intact-C (adj. *P* = 0.049), while differences between the other meals were not significant. Meal type had similar effects on the duodenal maltotriose concentrations (main effect *P* = 0.018, Broken versus Intact-C, mean difference 0.504 ± 0.473 mg ml^−1^, 410% higher, adj. *P* = 0.038) and duodenal glucose concentrations (main effect *P* = 0.003, Broken versus Intact-C, mean difference 0.253 ± 0.173 mg ml^−1^, 132% higher, adj. *P* = 0.007) as shown in Fig. 5c,d and Extended Data Fig. 3. Sucrose (liquid-phase meal marker) was present at ~1.4 × higher concentrations in the gastric fluid compared with the duodenal fluid, and duodenal sucrose concentrations were significantly different between meal types (*P* = 0.041), with Broken having a 0.080 ± 0.074 (90%) higher sucrose concentration than Intact-C (adj. *P* = 0.035); this could suggest a greater dilution of the Intact-C meals. No significant differences in duodenal sucrose concentrations were seen between Intact-S and Broken. Thus, the differences in maltose concentrations observed within these gastric or duodenal fluids after Intact-S and Broken can be taken to mainly reflect differences in meal starch digestibility, whereas Intact-C results may also be influenced by gastric emptying or dilution effects.

Pellet masses contained the inaccessible and undigested meal components from the aspirated gastric and duodenal fluids. Typically, 1 ml of digesta aspirated from the stomach contained between 7 and 48 mg of pellet dry mass, and duodenal aspirates contained between 3 and 18 mg of pellet dry mass (Supplementary Table 5 provides further details).

Regardless of meal type, the duodenal pellets consisted of ~43–54% ‘meal-derived’ carbohydrate, of which starch was the main component (Fig. 5e). There was a highly significant main meal effect (*P* < 0.001) on duodenal pellet carbohydrate composition, with duodenal pellets from Broken comprising a 30.54 ± 6.47 mg per 100 mg (46%) lower proportion of starch than Intact-S (adj. *P* < 0.001) and 33.01 ± 9.00 mg per 100 mg (43%) lower than Intact-C (adj. *P* < 0.001). Taking into account the typical pellet masses for each meal, it is possible to estimate that duodenal fluid after Intact-S, Intact-C and Broken (0–3 h) contained on average 6.353 ± 2.678, 3.263 ± 1.667 and 1.306 ± 0.456 mg non-bioaccessible starch per ml aspirated duodenal fluid (Fig. 5f). Based on the microstructure of the digesta (Fig. 4), it is likely that the higher levels of non-bioaccessible starch in digesta from the cellular meals are due to its encapsulation within the cell wall (type 1 resistant starch).

Overall, the cellular meals showed similar digestion properties, whereas the non-cellular Broken meal resulted in less undigested starch in the pellet, while having higher local maltose and maltotriose concentrations. Our in vivo observations support the cell wall barrier mechanism proposed for in vitro studies^37–40^ that the intact cell wall is the key to controlling starch digestion.

The oral processing of digestion deserves more attention. The role of salivary amylase is often overlooked due to the short duration of the oral phase. Our study suggested that disrupting the intact cell wall (‘Broken’ meal) increased starch digestibility in the mouth, which caused local concentrations of starch digestion products (maltose > maltotriose > glucose) to increase rapidly within the stomach and duodenum within the early-postprandial period after a Broken meal consumption. Additionally, we noted that the gastric pH remained at around 4 for the first 15–30 min after meals (Supplementary Table 6), likely allowing salivary amylase to retain 50% of its activity^41^. Our observations thus imply that salivary amylase digestion of accessible starch may continue in the stomach in humans and that the extent to which this contributes to overall glucose release from starch depends on meal cellular structure. We recommend future in vitro simulated digestion models incorporate the oral digestion phase and account for dynamic changes in gastric pH.

### Gastric maltose drives early glucose and incretin responses

**Extended Data Fig. 4:**
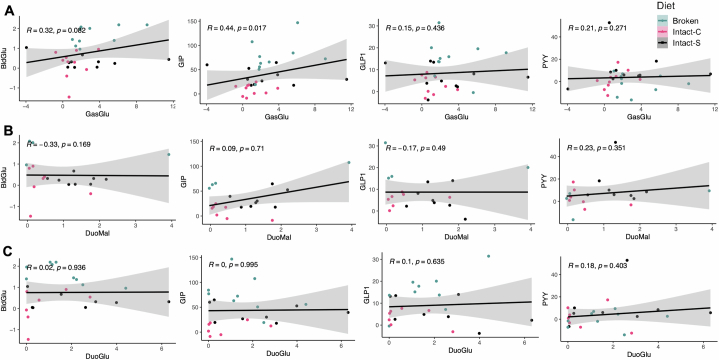
4 Spearman correlations of 0–30 min delta changes in luminal carbohydrates with blood responses. (**A**) Relationship between gastric glucose (mg/mL) and blood glucose (mmol/L), GIP(pg/mL), GLP-1 (pmol/L), and PYY (pmol/L). (**B**) Relationship between duodenal maltose (mg/mL) and blood glucose (mmol/L), GIP(pg/mL), GLP-1 (pmol/L), and PYY (pmol/L). (**C**) Relationship between duodenal glucose (mg/mL) and blood glucose (mmol/L), GIP(pg/mL), GLP-1 (pmol/L), and PYY (pmol/L). Two-sided Spearman correlation tests were used for scatterplots ABC. All scatterplots displayed individual points with a regression line representing the mean predicted values, and shaded bands representing 95% CI (n = 9). BldGlu, blood glucose; GasGlu, gastric glucose; DuoGlu, duodenal glucose; DuoMal, duodenal maltose. Source data

Early peaks in blood responses (0–30 min) were tightly correlated with initial rates of starch digestion (Fig. 5g). Correlations analyses were performed between the 0–30 min delta changes in blood responses with increments of starch digestion products: gastric glucose (GasGlu), gastric maltose (GasMal), duodenal glucose (DuoGlu) and duodenal maltose (DuoMal) across all meal types. GasMal emerged as the most robust correlator, positively correlating with elevations in glucose, GIP and GLP-1 levels (Fig. 5g; Spearman *ρ* = 0.64, 0.47, 0.42 with *P* < 0.01, *P* = 0.014 and *P* = 0.034, respectively). GasGlu played similar but less-pronounced correlations (Extended Data Fig. 4; *ρ* = 0.32, *P* = 0.082 for blood glucose, *ρ* = 0.44, *P* = 0.017 for GIP). No significant correlation was found between DuoGlu or DuoMal with blood responses (all *P* > 0.05; Extended Data Fig. 4), possibly due to dilution effects of gastric fluid entering the duodenum and/or limited duodenal samples (Supplementary Table 3 provides an overview of sample collection and analyses). No correlation was found between PYY and luminal starch digestion products (Extended Data Fig. 4; all *P* > 0.05).

Our data demonstrated a direct relationship between cell intactness, intestinal carbohydrates and the magnitude of the glycaemic response. The observation that spikes in blood glucose and GIP within 0–30 min were associated with increments of gastric carbohydrates is an interesting finding. This can be supported by previous studies that identified SGLT-1 and GLUT-1, the main glucose transporters^42,43^ as well as the GIP-positive K cells^44^ in the human and mouse stomach. Mechanisms of carbohydrate absorption and GIP-sensing are well described in the small intestine^45–47^, whereas our study provides additional insight into the early stage of digestion (the oral and gastric phase) and suggests that these can play a more important role in postprandial glucose and GIP than previously thought.

Our results also showed that the initial starch digestion rates correlated with GLP-1 but not PYY, which aligns with previous observations. The upper segment of mice intestines could secrete GLP-1 but not PYY^48^. Intragastric or intraduodenal glucose infusion in humans resulted in a notable increase in GLP-1 but had a lesser impact on PYY secretions^49,50^. Our findings supported the role of gastric and duodenal carbohydrates in the early GLP-1 response with minor effects on PYY.

### Duodenal undigested starch associated with PYY

We then explored whether the PYY response was a result of carbohydrates arriving at the lower gut (for example, the jejunum and ileum) where higher densities of L cells are present. The undigested starch contents in duodenal aspirates, indicating the availability of carbohydrates in the lower gut, positively correlated with PYY iAUC (0–180 min) (*ρ* = 0.39, *P* = 0.048; Fig. 5h). Non-digestible carbohydrates have been reported to arrive in the ileum as early as the first 30 min after meals in healthy participants^51^; ileal infusion of glucose increased PYY, whereas no such effects were observed with duodenal infusion^28^. These findings supported our analyses indicating that the undigested starch escaping to the lower intestinal segment promoted PYY secretion.

The undigested starch showed no correlation with the iAUC of GLP-1 or GIP within 0–180 min (data not shown). However, it exhibited significant correlations with their iAUC from 30 to 180 min (*ρ* = 0.62 and 0.62, both *P* < 0.001, as shown in Fig. 5h). Thus, carbohydrate arrival in the lower intestinal segment promoted PYY and later-phase GLP-1 responses.

### Intestinal digestion products, gut hormones and appetite

**Extended Data Fig. 5:**
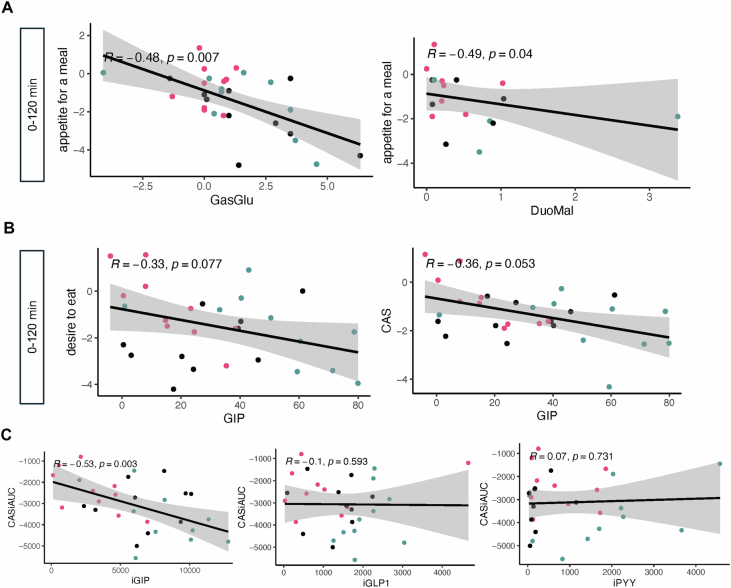
Spearman correlations between intestinal digestion products, gut hormones and satiety measurments. (**A**) Relationship between 0–120 min delta changes of gastric glucose (mg/mL) and duodenal maltose (mg/mL) and appetite for a meal (mm), n = 9. (**B**) Relationship between 0–120 min delta changes of GIP (pg/mL) and desire to eat (mm) and composite appetite scores (mm), n = 10. (**C**) Relationship between GIP iAUC pg/mL * (0–180 min), GLP-1 iAUC pmol/L* (0–180 min), PYY iAUC pmol/L* (pmol/L) and composite appetite scores mm*(0–180 min), n = 10. Two-sided Spearman correlation tests were used for scatterplots ABC. All scatter-plot displayed individual points with a regression line representing the mean predicted values, and shaded bands representing 95% CI. CAS, composite appetite scores; DuoMal, duodenal maltose; GasGlu, gastric glucose. Source data

We next analysed the association between starch digestion products, gut hormones and appetite VAS measurements. We focused on the 0–120 min delta changes where the most significant differences in gut hormones and satiety levels among meals were observed. The delta changes of DuoMal and GasGlu from 0 to 120 min showed a negative correlation with appetite for a meal (*ρ* = −0.49, *P* = 0.04; *ρ* = −0.48, *P* = 0.007; Extended Data Fig. 5a); suggesting the prolonged release of carbohydrate digestion products might reduce appetite.

GIP exhibited a negative trend with desire to eat and composite appetite with borderline significance (*ρ* = −0.33 and −0.35, *P* = 0.08 and 0.05; Extended Data Fig. 5b), whereas no significant associations were observed between satiety levels and GLP-1 or PYY (*P* > 0.05). This trend was consistent with their 0–180 min iAUCs, where GIP was associated with a reduction in composite appetite (*ρ* = −0.53, *ρ* = 0.003), but PYY and GLP-1 were not (Extended Data Fig. 5c; *P* > 0.05). Although GLP-1 and PYY are widely recognized as appetite-suppressing hormones, both can be influenced by stress and mood^52,53^, potential confounding factors in this study given the gut intubation procedures. GIP, co-expressed with GLP-1, has more limited and indirect associations with emotional states, potentially allowing its association with appetite suppression to persist.

### Food structure shaped gastric and duodenal metabolites

**Fig. 6:**
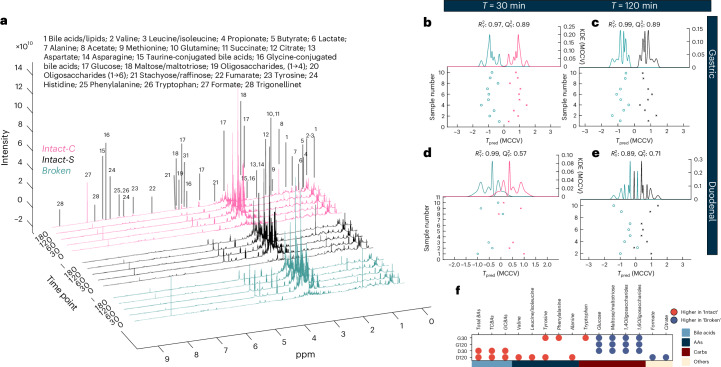
Food structure impacted gastric and duodenal metabolomics. **a**, Overviewing mean ^1^H-NMR spectrum of duodenal aspirates with annotations for peak assignments. Chemical shifts for NMR peak assignments are reported in Supplementary Table 7. **b**–**e**, RM-MCCV-PLS-DA models on ^1^H-NMR spectra derived from gastric and duodenal aspirates comparing participants receiving Broken and Intact-S/Intact-C meal at postprandial 30 and 120 min. Dots represent individual participant metabolic profiles. *Q*^2^Y, capability of prediction; *R*^2^Y, explained variance. Model scores: gastric postprandial (**b**) *T* = 30 (*n* = 9, *R*^2^Y 0.97, *Q*^2^Y 0.89). Gastric postprandial (**c**) *T* = 120 (*n* = 9, R^2^Y 0.99, Q^2^Y 0.89). Duodenal postprandial (**d**) *T* = 30 (n = 5, *R*^2^Y 0.99, *Q*^2^Y 0.57). Duodenal postprandial (**e**) *T* = 120 (*n* = 8, *R*^2^Y 0.89, *Q*^2^Y 0.71). **f**, List of luminal metabolites that were significantly associated with food structure interventions at postprandial 30 and 120 min (adj. *P* values < 0.05). *P* values were adjusted for multiple testing using the Benjamini–Hochberg false discovery rate (FDR) and reported in Supplementary Table 8. G30, gastric *T* = 30 min; G120, gastric *T* = 120 min; D30, duodenal *T* = 30 min; D120, duodenal *T* = 120 min; BA, bile acid; TCBA, taurine-conjugated BA; GCBA, glycine-conjugated BA.

Enteroendocrine cells (EECs) secrete gut hormones in response to metabolites in the gut lumen^22,54^. EECs and receptors have been well mapped throughout the gut^55^, yet little is known about how meal ingestion and transit influence the luminal metabolites. This study used ^1^H-NMR metabolomic profiling to measure the metabolomics in gastric and duodenal aspirates collected pre- and postprandially at 30, 60, 120 and 180 min. An overview of profiling and peak assignments is presented in Fig. 6a and Supplementary Table 7.

**Extended Data Fig. 6:**
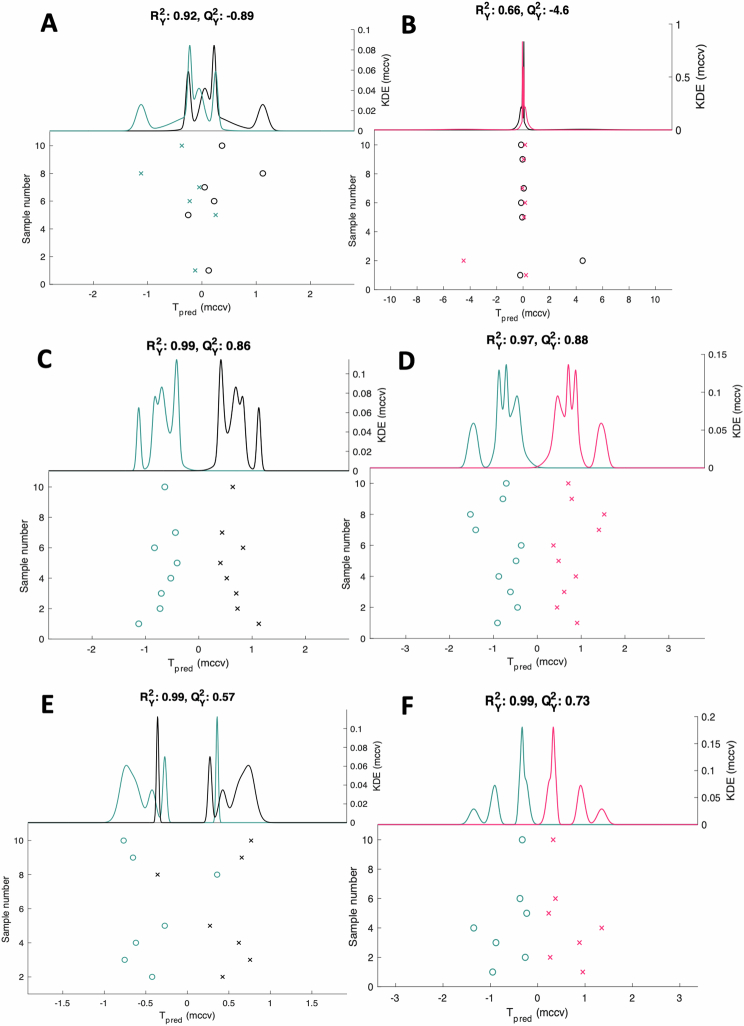
Metabolomic profiling of gastric and duodenal aspirates. Repeated-Measures, Monte Carlo Cross-Validation, Partial Least Squares Discriminant Analysis (RM-MCCV-PLS-DA) models on ^1^H-NMR spectra derived from gastric and duodenal aspirates comparing participants receiving Broken, Intact-S, or Intact-C meal pre- and postprandial 60 min. Dots represent individual participant metabolic profiles. Abbreviations: Q^2^Y, capability of prediction; R^2^Y, explained variance. Model scores: (**A**) Intact-S vs. Broken, gastric T = 0 (n = 6, R^2^Y 0.92, Q^2^Y −0.89). (**B**) Intact-S vs. Intact-C, gastric T = 0 (n = 7, R^2^Y 0.66, Q^2^Y −4.60). (**C**) Intact-S vs. Broken, gastric postprandial T = 60 min (n = 8, R^2^Y 0.99, Q^2^Y 0.86). (**D**) Intact-C vs. Broken, gastric postprandial T = 60 min (n = 10, R^2^Y Y 0.97, Q^2^Y 0.88). (**E**) Intact-S vs. Broken, duodenal postprandial T = 60 min (n = 7, R^2^Y 0.99, Q^2^Y 0.57). (**F**) Intact-C vs. Broken, duodenal postprandial T = 60 min (n = 7, R^2^Y 0.99, Q^2^Y 0.73).

RM, Monte Carlo cross-validation (MCCV), partial least squares discriminant analysis (PLS-DA) identified the metabolites associated with food structure interventions. No baseline differences between meals were detected (Extended Data Fig. 6). Robust separations between the ‘Intact’ meal and ‘Broken’ meal were observed between postprandial 30–120 min (30 min and 120 min in Fig. 6b–e and others in Extended Data Fig. 6).

At postprandial 30 min, when GIP and GLP-1 levels peaked for Broken and were lowest for Intact-C, robust differences in their luminal metabolites were observed (gastric model: *R*^2^Y 0.97, *Q*^2^Y 0.89; duodenal model: *R*^2^Y 0.99, *Q*^2^Y 0.57). Broken exhibited increased levels of starch digestion products, including maltose, glucose and oligosaccharides in gastric and duodenal aspirates. Broken showed decreased levels of tyrosine, phenylalanine and tryptophan in gastric aspirates, and decreased conjugated bile acids in duodenal aspirates compared with Intact-C (Fig. 6f; *P* values reported in Supplementary Table 8).

At postprandial 120 min, GLP-1 levels in Intact-S significantly surpassed those in Broken, with distinct differences observed in their gastric and duodenal metabolomes (gastric model: *R*^2^Y 0.99, *Q*^2^Y 0.89; duodenal model: *R*^2^Y 0.89, *Q*^2^Y 0.71). Intact-S exhibited elevated levels of amino acids and bile acids in duodenal aspirates, including valine, leucine/isoleucine, alanine, tyrosine and taurine/glycine-conjugated bile acids. Although starch digestion products in gastric aspirates remained lower in Intact-S compared with Broken, these carbohydrates were comparable in their duodenal aspirates (Fig. 6f; *P* values reported in Supplementary Table 8).

### Intestinal metabolite dynamics and postprandial responses

**Fig. 7:**
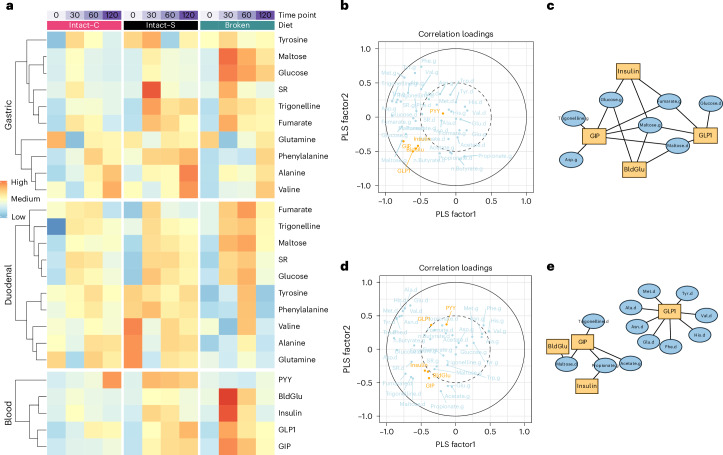
Interplay between luminal metabolites and blood metabolic responses. **a**, Dynamics of luminal metabolites and blood responses. Each block represented the mean concentrations of participants (*n* = 10), Each variable was normalized using *z*-score transformation. SR, stachyose/raffinose. **b**,**d**, PLSR model correlation loadings for metabolites and blood responses at postprandial 30 and 120 min. Metabolites and associated blood responses were analysed via PLSR models (*x* (metabolites)/*y* (blood responses), *n* = 10). Positively correlated variables are proximal within the correlation circle (angle <0), while negative correlations appear diametrically opposed (angle <180). Variables lacking correlation are situated at angles <90 relative to each other. The *x* and *y* axes denote the explained variance percentages by the PLSR factor. **c**,**e**, Correlation networks showing significant correlations between *x* (metabolites) and *y* (blood responses) at postprandial 30 and 120 min (*n* = 10), using an FDR-adjusted *P* value threshold of <0.05 using the Benjamini–Hochberg method. Gastric and duodenal metabolites are indicated with a suffix ‘.g’ or ‘.d’. Standard three-letter abbreviations are used for amino acids. Source data

Figure 7a overviews how luminal metabolites change over time and correspond with blood responses. Key metabolites were quantified from their ^1^H-NMR profiling and presented in a heatmap. Consistent with the quantitative analyses, this figure also shows that cell breakage increased glucose and maltose concentrations within the stomach and the duodenum, which coincided with higher blood glucose, insulin, GLP-1 and GIP concentrations. Time-series comparison of meal-derived components such as trigonelline (a marker of legume intake^56^), stachyose/raffinose (a source from legume meal), fumarate/fumaric acid (a food additive from the background diet jelly) with starch digestion products (maltose and glucose) confirmed that the elevated levels of starch digestion products coincided with meal transit.

Amino acids such as alanine, tyrosine and glutamine were more concentrated in the fasted state and decreased postprandially, aligning with a dilution effect from postprandial intestinal secretions and meal transit. The duodenal amino acid concentrations rose in the later postprandial period (60 and 120 min), with the increase being more pronounced for the Intact-cellular meals and concurring with their later GIP /GLP-1 response.

Partial least squares regression (PLSR) explored the relationships between luminal metabolites (X matrix) and blood responses (Y matrix). PLSR models for early and later postprandial periods revealed a shift of luminal metabolites and enteroendocrine signalling. At postprandial 30 min, Fig. 7b showed a distinct clustering of blood glucose (BldGlu), insulin, GIP and GLP-1 alongside gastric maltose. The network correlation analysis (Fig. 7c) highlighted the significant correlations (with FDR-adjusted *P* values < 0.05), involving maltose, glucose, fumarate and trigonelline in the stomach.

In the later postprandial stage, BldGlu, insulin and GIP were most closely associated with duodenal maltose, trigonelline and fumarate (Fig. 7d). GLP-1 showed the same vector direction with a series of duodenal amino acids (Fig. 7d). Among these were valine, alanine, glutamine, tyrosine and histamine, highlighted as significant correlators (Fig. 7e).

Food structure has been hypothesized to impact gut hormone secretion through modulating the amount of nutrients arriving in the distal intestine^5^. Less attention has been paid to the proximal intestine. This study provides insight into the upper gastrointestinal mechanisms by which Broken and Intact food structures induce different gut hormone responses. Gastric and duodenal metabolite profiles significantly differed between food structure interventions, with the differences driven by bile acids, amino acids and starch digestion products. Furthermore, the PLSR models demonstrated the dynamic associations between luminal metabolites and postprandial blood responses: initial glycaemia and GIP responses were predominantly influenced by a surge in maltose, particularly in the gastric region. In the later postprandial phase, the metabolic drivers shift towards a range of duodenal amino acids that have implications for GLP-1 levels. Specifically, we showed that elevated valine, alanine and tyrosine (Fig. 6e,f) may be linked to a heightened GLP-1 response in Intact-S at postprandial 120 min. Emerging evidence suggests individual amino acids can stimulate GLP-1 with different potencies and through different mechanisms^57^. Valine has been suggested as a potent stimulator of GLP-1 secretion when infused in the perfused rat small intestine^58^. In humans, intraduodenal infusion of l-tryptophan^59^ or l-glutamine^60^ induced a GLP-1 response; other amino acids have yet to be studied. Our data indicate that the relationship between the duodenal amino acid profile and GLP-1 response warrants further investigation. Further research is also needed to uncover the impact of food structure on luminal amino acid releases to complete the understanding of how chickpea meals containing intact cell walls promote GLP-1 secretion.

### Food structure and serum metabolites

We investigated whether serum metabolites were modified by diets in a similar way to intestinal metabolites. Serum metabolites were measured using ^1^H-NMR and their quantifications were exported from Bruker reports. Eleven mutual metabolites were identified in the serum and stomach/duodenum, including eight amino acids (alanine, glutamine, methionine, asparagine, tyrosine, phenylalanine, histidine and valine), glucose, acetate/acetic acid and formic acid/formate). We compared the serum metabolites between meals at 30 and 120 min, when different gastric and duodenal metabolic profiles between meals were observed.

Consistent with gastric and duodenal profiles, serum glucose at 30 min was higher in Broken compared with Intact-S and Intact-C (paired Wilcoxon signed-rank test with FDR-adjusted *P* values: Broken > Intact-C, *P* = 0.029; Broken > Intact-S; *P* = 0.029). At 120 min, we did not observe the elevated tyrosine, alanine or valine in Intact-S serum as seen in the duodenum compared with the Broken meal (all *P* > 0.05; Supplementary Table 10). Intact-S showed a trend toward increased serum methionine, although this was not statistically significant (FDR-adjusted *P* = 0.097). No significant differences were found in acetate and formate levels between diets in serum, stomach or duodenum (all *P* > 0.05; Supplementary Table 10).

**Extended Data Fig. 7:**
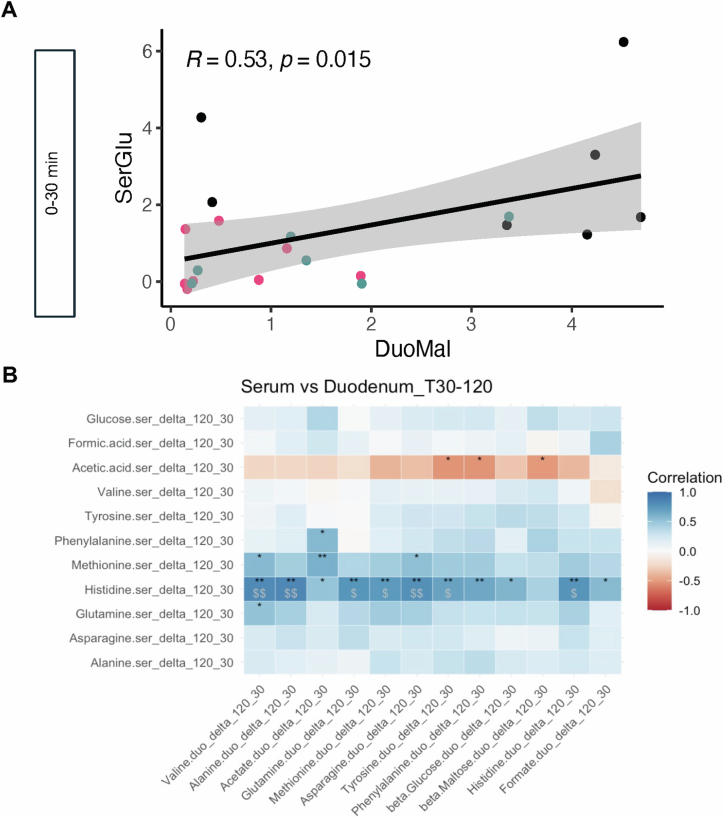
Spearman correlations between serum and duodenal metabolites. (**A**) Relationship between 0–30 min delta changes of duodenal maltose (mmol/L) and serum glucose (mmol/L). Two-sided Spearman correlation test was used.The scatter-plot displayed individual points with a regression line representing the mean predicted values, and shaded bands representing 95% CI (n = 10). (**B**) Spearman correlation matrix showing the relationships between 30–120 min delta changes of serum and duodenal metabolites. Metabolites include, formic acid, acetic acid, and a range of amino acids such as valine, tyrosine, phenylalanine, methionine, histidine, glutamine, asparagine, alanine in the serum (indicated by “_ser”) and duodenum (indicated by “_duo”). The colour scale represents the Spearman correlation coefficient, ranging from −1.0 (red, strong negative correlation) to +1.0 (blue, strong positive correlation). Two-sided Spearman correlation tests were used. P values before and after false discovery rate (FDR) adjustments are shown with asterisks and dollar signs: $*P < 0.05, $$**P < 0.01, and $$$***P < 0.001.n = 10. Source data

We examined correlations in serum and duodenal glucose within 0–30 min and correlations in amino acid pairs within 30–120 min delta changes, capturing their dynamics in the duodenum (as shown in Fig. 7a). A significant positive association was observed between duodenal maltose and serum glucose at 0–30 min (*ρ* = 0.53, *P* = 0.015; Extended Data Fig. 7a). At 30–120 min, serum histidine displayed a significant positive correlation with duodenal histidine and other duodenal amino acids (all FDR-adjusted *P* < 0.05; Extended Data Fig. 7b), while no other amino acid pairs showed significant correlations. Our ongoing work, comparing metabolites across the small intestine, suggests that the duodenum tends to be richer in carbohydrates; amino acid levels are relatively low in the duodenum and higher in the ileum. This may account for the stronger links observed between duodenal and serum carbohydrates and the weaker associations seen for amino acids, and highlights the importance of considering multi-regional intestinal absorption.

### Food structure did not affect serum FGF19 levels

**Extended Data Fig. 8:**
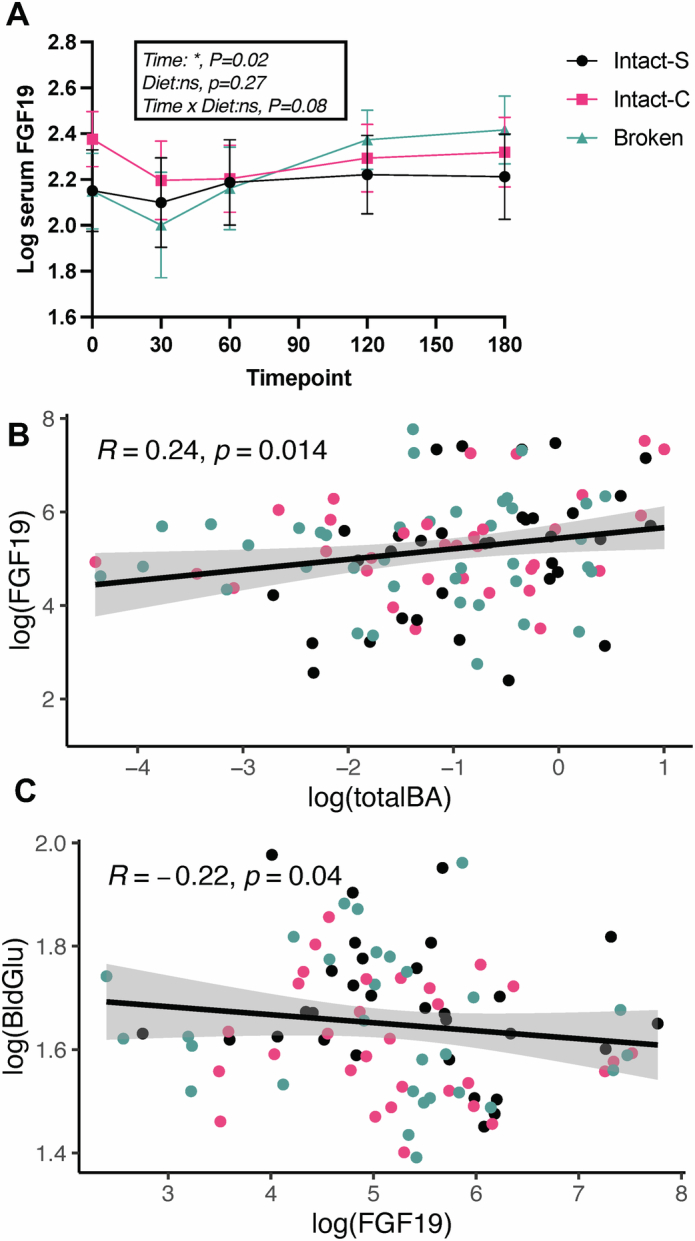
Serum FGF19 and association with duodenal bile acid and blood glucose. (**A**) Postprandial serum FGF19 levels (pg/ml, log-transformed) following Intact-S, Intact-C, and Broken meals. Two-way ANOVA indicated no significant effect of Diet (P = 0.27) and no significant Time × Diet interaction (P = 0.08). There was a significant effect of Time (P = 0.02). * P < 0.05. Data shows mean with SEM, n = 10. (**B**) Spearman correlation between duodenal total bile acids (mmol/L, log-transformed) and serum FGF19 (pg/ml, log-transformed). Two-side Spearman correlation test was used. The scatter-plot displayed individual points with a regression line representing the mean predicted values, and shaded bands representing 95% CI (n = 10). (**C**) Spearman correlations between blood glucose dip at T60–180 min (mmol/L, log-transformed) and serum FGF19 (pg/ml, log-transformed). Two-side Spearman correlation test was used. The scatter-plot displayed individual points with a regression line representing the mean predicted values, and shaded bands representing 95% CI (n = 10). Source data

FGF19, an intestinal hormone that regulates bile acid metabolism, showed no differences across meals (diet, *P* = 0.27, time × diet, *P* = 0.08; Extended Data Fig. 8a). A time-dependent increase was noted (time, *P* = 0.020) in the Broken meal at 120 and 180 min (adj. *P* = 0.048 and 0.026).

We explored the association between duodenal bile acids and serum FGF19 levels and observed a weak but significant correlation (*ρ* = 0.24, *P* = 0.014; Extended Data Fig. 8b). FGF19 is known to be stimulated by bile acid reabsorption in the ileum via Farnesoid X receptor (FXR) activation. While FXR mRNA has been detected in the duodenum of mice, it is found at much lower concentrations compared with the ileum^61,62^. There is currently no evidence to indicate whether FXR is present in the human duodenum. Thus, observing only a weak association between duodenal bile acids and FGF19 is not a surprise.

FGF19 has a regulatory effect on blood glucose levels, typically observed after insulin and incretin peaks^63^. We found a correlation between serum FGF19 and the dip of blood glucose over 60–180 min (*ρ* = −0.21, *P* = 0.01; Extended Data Fig. 8c), supporting its role in glucose metabolism in healthy individuals.^63^

## Discussion

Previous studies^19–21,64^ have shown that food structure has a profound effect on blood glycaemia and gut hormone responses. Our study supports these findings as we demonstrate chickpea meals with the same nutrient contents and contrasting cell intactness were shown to elicit significantly different postprandial blood glucose, insulin, GIP, GLP-1, PYY and satiety responses. However, the current study goes beyond these observations by exploring the relationship between the structure of the food matrix, luminal metabolites of the small intestine and the generation of signals that affect physiology.

We showed that disrupting the cellular structure (‘Broken’ meal) significantly increased starch bioaccessibility in the upper gastrointestinal tract, leading to a twofold to fourfold increase in the magnitude of the (peak) glucose response evoked. The ‘Broken’ meal also increased insulin and incretin release to combat the rapid rise in plasma glucose. Furthermore, the ‘Broken’ meal shortened the duration of the GIP, GLP-1 and PYY responses, and lowered subjective satiety compared with a nutrient-matched meal in which the cellular structure remained Intact (Intact-S). It is noteworthy that the physiological benefits for glycaemic control and weight loss, as reported in earlier epidemiological and interventional studies, were when whole cooked pulses, with their cellular structure presumed intact, were usually the main structural form consumed^65,66^. However, this study shows that these benefits can be compromised when plant cell intactness is disrupted. Food cellular structure and processing level should be considered when making dietary recommendations or developing health-promoting ingredients for fibre-rich products.

The nutrient-sensing system on EECs related to the release of gut hormones is a topic of growing interest. Unlike the ileum and colon, lesser attention has been paid to the proximal intestine about the secretions of anorexigenic gut hormones such as GLP-1. Our study emphasizes the role of the postprandial small intestinal metabolites, demonstrating how digestion of ‘Broken’ and ‘Intact-cellular’ meals shapes different upper gastrointestinal metabolite profiles, resulting in distinct endocrine and metabolic consequences. Significant alterations in metabolite profiles were observed, particularly in starch digestion products, amino acids and conjugated bile acids, offering new insights to explore the interaction between food and gut signalling. We showed the ‘Broken’ meal elicited a higher acute GIP and GLP-1 response as a consequence of a rapid rise of gastric maltose, whereas the ‘Intact-cellular’ meal elevated small intestinal tyrosine, valine and alanine, which were associated with prolonged GLP-1 levels. Future research should investigate the mechanisms underlying these findings and validate their roles in gut hormone secretion through well-controlled human gut infusion studies.

Pharmaceutical adaptions of gut hormones are creating a new generation of therapies for diabetes and obesity. Our study indicates that food structure can be a promising tool to target gut hormones by controlling the release and delivery of nutrients in the gut, which could offer a public health strategy to prevent non-communicable disease. Processing that disrupts the cellular structure and increases intestinal luminal maltose and glucose could have positive effects on K cell release of GIP, whereas intact structures and the change in metabolite profiles related to these structures lead to increased GLP-1 and PYY from L cells. Simple changes in food structure could therefore offer an effective strategy for optimizing gut peptides and postprandial metabolism.

### Strengths and limitations

A strength of this study was the use of precisely controlled meal structures in combination with the parallel sampling of blood and digesta from healthy humans within a controlled clinical environment. The meals were matched by the amount and type of carbohydrate, while clear differences in the cellular structure were achieved. This allowed meal structure-dependent differences to be elucidated. Insight into the gastrointestinal mechanisms that underpinned the different postprandial blood responses to meals with contrasting structures were possible through the use of an enteric intubation technique that enabled postprandial sampling of digesta in parallel to blood collections. This technique helps to overcome limitations of in vitro digestion models, which do not, for instance, replicate oral phase processing and enteroendocrine feedback responses.

This present study has a few limitations. A learning from this enteric intubation (pilot) study relates to the sampling frequency of postprandial duodenal digesta, as the low volume of digesta prevented some sample collections, resulting in insufficient sample numbers/volumes for reliable insight into digesta time trends. Future studies should consider less frequent postprandial sampling of stomach and duodenal content to improve sample collection. Sampling should expand to the distal gut area to develop a complete picture of the interactions between gut metabolites and hormones. Second, the threshold for significance was set at *P* < 0.05 in this study. Amrhein et al. have raised concerns that this threshold may be too low to indicate biological relevance^67^. Specifically, the differences in fullness between Intact-S and Intact-C (*P* = 0.031) and the iAUC GLP-1 between Intact-S and Broken (*P* = 0.020) should be interpreted with caution. Further work is required to verify whether these findings translate into significant clinical outcomes. A key focus should be to determine whether prolonged intake of Intact chickpea meals could improve long-term glycaemic outcomes (for example, homeostatic model assessment for insulin resistance and glycated haemoglobin) and weight loss. Last, it should be acknowledged that this is a pilot study with a short wash-out period and a small sample size. Although no significant differences were observed in fasting levels of glycaemia and gut hormones across our interventions, and a previous study^64^ using the same wash-out period reported no impacts on changes in gut microbiota composition due to food structures, carry-over effects on other metabolic functions cannot be completely ruled out.

## Methods

### Ethics statement

This trial was approved by the Health Research Authority and London-Camden and King’s Cross Research Ethics Committee (REC 19/LO/0962) before the commencement of any study procedures. The study was prospectively registered at ISRCTN (ISRCTN18097249) before the enrolment of the first participant. All the participants received a participants information sheet and signed informed consent before they started the clinical trial. The research has been performed in accordance with the Declaration of Helsinki. The trial protocol can be assessed in the Supplementary Text 2.

### Human intubation study design

A CONSORT diagram and key study dates are shown in Extended Data Fig. 1. A human study was conducted with ten healthy participants aged 18–65 years with a BMI of 18.5–30 kg m^−2^. All participants were recruited from the healthy volunteer database of NIHR Imperial CRF. Participants expressing an interest were asked to complete a pre-screening form and attend a screening visit. Their eligibility was checked and confirmed by a medical doctor based on their health history, anthropometric measurements, ECGs and blood test results. Participants with an abnormal ECG, screening blood values outside the clinical reference range, a history of cancer, diabetes, gastrointestinal disease and/or requiring medication likely to interfere with metabolic and hormone responses were excluded. Inclusion and exclusion criteria were as listed in Supplementary Table 1.

This study followed a double-blinded, randomized crossover design. Randomization was performed using a sealed envelope system (Sealed Envelope 2022). Participants were randomly assigned into three intervention groups using a balanced allocation ratio of 1:1:1. The randomization was performed by an independent researcher who was not involved in this trial. The allocation of treatment sequence was blinded to the investigators, the technicians performing analysis of blood samples and participants. Investigators and participants remained blinded until the completion of the study and data analysis.

The primary outcome was the blood gut hormone response. Co-secondary outcomes included intestinal content analysis, blood glucose and insulin response, subjective appetite changes and ab libitum energy intake.

### Study procedure

Each participant attended one 4-day inpatient study visit at NIHR Imperial CRF at Hammersmith Hospital. The day before the study visit, participants were asked to refrain from caffeine, alcohol and strenuous exercise. Participants were also requested to fast overnight. On day 1, an enteral feeding tube was placed in the participants’ small intestine (duodenum), following procedures previously reported^64^. On days 2, 3 and 4 participants received one of three chickpea test meals (see ‘Dietary interventions’) in a randomized order. Intestinal samples were taken before meals (*T* = −10 and 0 min) and subsequently at 15-min intervals for 180 min for microscopic and carbohydrate analyses. Blood samples were taken before and after the test meals for 180 min (*T* = −10, 0, 15, 30, 45, 60, 90, 120, 150 and 180 min). Blood samples were collected through a cannula placed in the antecubital fossa used for measuring blood glycaemia and hormonal markers. A VAS questionnaire was collected for the same period and the same intervals to measure the subjective appetite levels of participants. A lunchtime meal (at 4 h) was provided to measure their ad libitum food intake. On day 4, the enteral tube was removed and participants were discharged.

### Dietary interventions

Participants received different test meals on day 2, 3 and 4 corresponding to the three different chickpea structures: Broken, Intact-S and Intact-C. Each test meal contained identical ingredients and macronutrient composition per serving but differed in microstructure. See Supplementary Text 1 for full preparation protocol and compositional details.

### Sample analysis

#### Blood biochemical analysis

Blood samples were collected into tubes (BD Vacutainer tubes: fluoride/oxalate tubes for glucose analysis; SST serum tubes for insulin and metabolite analysis) and into lithium heparin tubes with DPP-IV (10 µl ml^−1^ blood, Merck Millipore), aprotinin (10,000 kIU ml^−1^ blood, Nordic Pharma) and AEBSF (pefabloc, 1 mg ml^−1^ blood) for GIP, GLP-1 and PYY analysis. Plasma glucose was measured using GLUC-PAP kits (Randox Laboratories). Serum insulin concentrations were determined by a Human Insulin Specific Radioimmunoassay (RIA) kit (HI-14K, Merck). Plasma GIP concentrations were measured by Human GIP ELISA kits from (EZHGIP-54K, Merck). All these assays were performed as per the manufacturers’ instructions. GLP-1 and PYY concentrations were measured by the in-house RIA method^68^. Serum FGF19 concentrations were measured using Human FGF19 ELISA kit (EHFGF19, Thermo Fisher Scientific). Serum samples were thawed and centrifuged for 10 min at 3,000*g* at 4 °C. Then, 350 μl of serum mixed with 350 μl of phosphate buffer (75 mM Na_2_HPO_4_, 2 mM NaN_3_ and 4.6 mM sodium trimethylsilyl propionate-[2,2,3,3-2H4] (TSP) in D_2_O, pH 7.4 ± 0.1). Then, 600 μl of the mixture was used for ^1^H-NMR metabolite quantification. A full quantitative calibration was completed before the analysis using a previously described protocol^69^ (Supplementary Text 3 provides details).

#### Gastric and duodenal starch digestion analysis

Defrosted gastric and duodenal samples were centrifuged for 15 min at 3,000*g* to separate the undigested solids (pellet) from the intestinal fluid. The supernatants were decanted into 15-ml centrifuge tubes. Absolute ethanol was added to the supernatant (3 ml) and pellet (1 ml) to kill bacteria before drying the sample fractions at 51 °C for 3 h in a centrifugal evaporator (EZ-2 Elite, Genevac). The dried supernatants were resuspended in 0.5 ml water (aided by vortex mixing and sonication), and then centrifuged at 13,000*g* for 5 min. The supernatants were diluted in (milliQ) water and 45 μl of each sample was transferred to an HPLC vial and 5 μl d-glucose-^13^C_6_-Glc 99% atom C (0.1 mg ml^−1^) was added as an internal standard, before analysis by LC–MS (Agilent 6490 mass spectroscopy) on a reverse-phase UPLC column (Thermo Hypercarb 100 × 2.1 mm 3-μm column) using 0.1% formic acid in water and 0.1% formic acid in acetonitrile as the mobile phase. Maltose, sucrose and maltotriose were included as standards. Sugars were quantified by selective ion monitoring. Gastric and duodenal glucose was measured by a GLUC-PAP kit (Randox Laboratories) based on the instructions, except for an additional step at the beginning. Defrosted samples were first centrifuged at room temperature for 10 min at 3,000*g*. A volume of 20 µl from the supernatant was used to perform the remaining steps according to the GLUC-PAP kit instructions.

Pellets were analysed for estimation of their carbohydrate composition: samples of the dry pellet powders <3 mg were weighed out to an accuracy of 0.1 mg into glass culture tubes, then treated with 100 μl 72% w/w H_2_SO_4_ for 3 h at room temperature, then diluted with water to 4% acid, and heated at 121 °C for 1 h in a Techne Dri-block heater, and finally cooled on ice for 10 min (ref. ^70^). Monosaccharide analyses of the acid hydrolysates were performed^71^. A mixed standard solution containing 200 μg ml^−1^ of nine monosaccharides (arabinose, fucose, galactose, glucose, galacturonic acid, glucuronic acid, mannose, rhamnose and xylose) was prepared and diluted to concentrations of 160, 120, 80 and 40 μg ml^−1^. Next, 100–300 µl of 1 mg ml^−1^ Talose internal standard) was added to each hydrolysate and standards mixture. Sample hydrolysates were then pH neutralized with 2 M CaCO_3_ and centrifuged (1,500*g*, 10 min) to remove the precipitate. The supernatants were filtered through 0.45-μm syringe filters. Finally, samples plus 5 µl d-glucose-^13^C_6_, 99% Atom C (0.1 mg ml^−1^), were derivatized with 3-methyl-1-phenyl-2-pyrazoline-5-one (PMP) and the monosaccharides quantified by UPLC analyses (Agilent 6490 Mass Spectrometer) using the method of Xu et al. (2018)^71^. Sample dry matter (g ml^−1^) was calculated from the pellet mass loss upon drying. The total monosaccharide content of the acid hydrolysates (derived from cell walls and starch) was calculated as the sum of anhydro masses of monosaccharide constituents and expressed per mg pellet dry mass. An indication of the amount of starch in the pellet samples was obtained as per equation (1). The glucose measured derives from starch, cellulose and xyloglucan. By acid hydrolysis of the chickpea cell wall purified of intracellular contents^72^, the ratio of arabinose to glucose was found to be 1:0.469. Assuming no solubilization or fermentation of pectic arabinan in the upper gut, the arabinose value may be used to estimate the non-starch glucan content. This was subtracted from the total glucose to give an indication of the starch content of the pellets.

1 $${Starch} \% ={Glu} \% -(0.469\,\times{Ara} \% )\times{\rm{Total}}\; {\rm{sugars}}\;({\mu{\rm{g}}}/{\rm{mg}}\; {\rm{dry}}\;{\rm{matter}})$$

The proportion of pellet total sugars that is derived from starch (starch %) is estimated from the proportion of glucose (Glu) and arabinose (Ara) measured in the acid hydrolysate, which are expressed as a percentage of the total sugar content (sum of anhydro masses of monosaccharide constituents) within the acid hydrolysate.

Concentrations of glucose, maltose and maltotriose, as the main starch digestion products, were plotted on time-series profiles. The calculation of iAUC for some participants was not feasible due to missing data (Extended Data Fig. 1 provides an overview of sample collection and analyses), so concentrations were averaged over the entire postprandial period to gain insight into the effect of meal structure on intestinal contents.

#### Structural analyses by light microscopy

An Olympus BX53 Upright Microscope equipped with CellSens software was used to analyse the structures of gastric and duodenal aspirated samples. All aspirates collected from each participant were imaged a minimum of ten times at different magnifications. In total, over 5,700 images were acquired, from which representative images were selected and presented in Fig. 4.

#### Gastric and duodenal metabolomic analysis

##### Metabolite extraction

Metabolites were extracted from gastric and duodenal aspirations^33^. Defrosted samples were centrifuged at room temperature for 15 min at 3,000*g*. Then, 450 μl of supernatant was mixed with 1 ml methanol, 2 ml chloroform and 1 ml water sequentially. The mixture was vortexed, centrifuged at room temperature for 15 min at 3,000*g*, and separated into aqueous and the organic phases. The aqueous phase was evaporated by a speed vacuum concentrator (Eppendorf Concentrator Plus) to dryness for ^1^H-NMR spectroscopy.

##### ^1^H-NMR spectroscopy

The aqueous phase of gut samples was re-constituted in 640 μl of H_2_O and sonicated for 10 min. Samples were checked after sonication and there was no visible precipitation. Then, 60 μl of NMR buffer (1.5 M KH_2_PO_4_ buffer (pH 7.4, 100% D_2_O, 2 mM sodium azide and 1 mM TSP (3-trimethylsilyl-[2,2,3,3,-^2^H_4_]-propionic acid sodium salt)) was mixed with 540 μl of samples and the mixture was further transferred to 5-mm NMR tubes. Quality controls were prepared by pooling 100 μl of each sample, independently for gastric and duodenal samples.

^1^H-NMR spectroscopy was performed at 300 K on a Bruker 600 MHz spectrometer^69^ (Bruker Biospin). It followed a standard one-dimensional pulse sequence with saturation of the water resonance RD – gz, 1 – 90° – *t* – 90° – *t*_m_ – gz, 2 – 90° – ACQ (noesygppr1d), where RD is the relaxation delay, gz is the z-gradient, 90° represents the applied 90° radio frequency (rf) pulse, *t* is an interpulse delay set to a fixed interval of 4 μs, RD was 2 s and *t*_m_ (mixing time) was 100 ms. Water suppression was achieved through irradiation on the water signal during RD and *t*_m_. Each 21 spectrum was acquired using four dummy scans followed by 64 scans and collected into 64,000 data points. The receiver gain was 90.5. A spectral width of 20,000 Hz was used for all the samples. Before Fourier transformation, the free induction decays (FIDs) were multiplied by an exponential function corresponding to a line broadening of 0.3 Hz.

A two-dimensional ^1^H−^1^H J-resolved experiment was also acquired to detect the J-couplings in the second dimension using the pulse programme with suppression of the water resonance during the RD (jresgpprqf). The following acquisition parameters were used: 16 dummy scans and two scans, 8,000 points with spectral window of 16.7 ppm for f2 and 40 increments with a spectral window of 78 Hz for f1, incremented delay of 3 μs, RD of 2 s and ACQ of 0.41 s. The receiver gain was set to 90.5.

##### NMR data processing

Multivariate statistical analysis was performed on spectra data. Each spectrum (~24,000 spectral variables) was automatically phased, baseline corrected, digitized over δ −0.5 to 10 and imported into MATLAB (R2021a, MathWorks), using in-house scripts. Spectral regions corresponding to the internal standard (δ −0.5 to 0.5) and water (δ 4.7 to 4.9) were excluded.

##### Metabolite identification

Following a published protocol^33^, statistical spectroscopic tools such as statistical total correlation spectroscopy (STOCSY) and subset optimization by reference matching (STORM) were used to identify spectra signals. Internal and external databases such as the Human Metabolome Database (HMDB; http://hmdb.ca/) and the Biological Magnetic Resonance Data Bank (https://bmrb.io/) were used for confirmation of assignments. Identified metabolites with the corresponding NMR spectra ppm values are listed in Supplementary Table 7.

##### Metabolite quantification

Quantification of metabolites was achieved using in-house developed routines written in both JavaScript and R. NMR spectra were processed according to the Bruker IVDr standard procedures^69^. No further processing was deemed necessary. Normalization is not required before quantification and can be applied on the extracted concentrations when necessary. The metabolites of interest were grouped in spectral regions of interest (SROI). For each SROI, a list of signals was established with attributes (parameters) that define their line shape (chemical shift, linewidth, multiplicity and scalar coupling). Those initial guesses were used as input for a gradient descent optimization (Levenberg–Marquardt) algorithm. All resulting models (pseudo-Voigt) were graded using a simple heuristic. Models with lower grades were visually inspected using a suite of interactive tools build in JavaScript (Supplementary Fig. 1). The modelled area under the curves were then converted to concentrations using the ERETIC internal standard and calibration procedure that is part of the Bruker IVDr protocol. When possible, correlations between concentration values integrated from distinct signals of the same metabolite were evaluated to validate the procedure (Supplementary Fig. 2).

### Appetite and food intake analysis

The VAS consisted of a set of questions to measure hunger (“how hungry do you feel right now?”), fullness (“How full do you feel right now?”), desire to eat (“How strong is your desire to eat”) and prospective food intake (“How strong is your appetite for a meal?”). Participants were asked to answer these questions by drawing a vertical line across a 100-mm scale ranging from “not at all” (right extreme) and “extremely” (left extreme). A composite appetite score was calculated by combining the four measurements (hunger + (100 − fullness) + desire to eat + appetite for a meal) / 4. An excessive, homogenous pasta meal was served at 240 min to measure ad libitum food intake. The meal consisted of 3 kg boiled white pasta, mixed thoroughly with two pots of tomato sauce (Hearty Food Co. Tomato Herb Pasta Sauce 440 g) and 50 g of vegetable oil (Flora sunflower oil) to provide approximately 2,500 kcal. Food intake was measured by weighing the food before and after consumption and calculating the difference.

### Sample size and statistical analysis

#### Sample size calculation

This was a pilot study. No similar study had been conducted before this study so the target of 15 participants was estimated. During the study, Petropoulou et al. used a similar methodology to investigate the effects of resistant starch from peas on human gastric and duodenal digestion with ten participants and reported significant differences on outcome measures such as blood glycaemia, gut hormone and intestinal starch digestion^64^. Thus, ten participants were recruited for this study.

Prospective sample size calculations were performed using G*Power (v.3.1.9.6). Based on Dagbasi et al., postprandial PYY measurements at 120 min between the LF and D-HF groups indicated an effect size of 1.34 (mean difference 11.54, s.d. = 8.62)^73^. With an *α* level of 0.05 and a power of 0.8, a sample size of seven was required. Additionally, Petropoulou et al. showed that small intestinal glucose measurements between RR and rr peas indicated an effect size of 0.87 (mean difference 145.55, s.d. = 167.11)^64^, which required a sample size of ten at an *α* level of 0.05 and a power of 0.8. Thus this study with ten participants had sufficient statistical power to observe differences in our primary outcome postprandial gut hormones and secondary outcome intestinal glucose levels.

#### Statistical analysis

Data analysis was performed blind to the interventions, with no data points excluded. Data were analysed using GraphPad Prism (v.9.0, GraphPad Software USA, Biomatters), R Studio (v.1.4.1106, R Core Team) and MATLAB (R2021a, The MathWorks). Data were checked for normality by the Shapiro–Wilk test. When data did not pass the test (*P* < 0.05), a log transformation was applied before conducting parametric tests.

For blood biochemical and subjective appetite data, two measurements of baselines before breakfast were combined as the baseline value. Differences between groups were assessed using repeated measure two-way ANOVA, followed by post hoc Tukey’s Tests for pairwise comparisons. The maximum rise from fasting concentrations, iPeak and the iAUC was analysed by one-way repeated measure ANOVA with post hoc Tukey’s test. The mean differences (MDs) with 95% CI were reported in the text (expressed as MD ± 95% margin of error). Results were considered statistically significant at *P* < 0.05.

For intestinal content data, due to many missing values, specific statistical methods were applied. For intestinal starch product analysis (Fig. 5a–f), a two-way mixed-effects ANOVA was originally planned for the time-series. However, the large number of missing values (due to potentially non-random variable availability of intestinal content) rendered this approach unreliable. As a result, time-series data are presented as mean data and Supplementary Table 3 includes a table showing number of samples used to compute the means for each time point. Mean analyte concentrations in samples collected from each participant over the 3-h period were analysed by one-way RM ANOVA, to test for main meal effects. Tukey’s test was performed post hoc when significant main effects were observed. Mean difference with 95% CI of difference, along with multiplicity adjusted *P* values are reported for post hoc pairwise comparisons. Spearman correlation analysis was performed to assess relationships between intestinal contents and blood glucose, GIP and GLP-1 (Fig. 5g–f).

For intestinal metabolomic analysis (Fig. 6b–e), data were centred and scaled to account for the RM design and then modelled using PLS-DA with MCCV^74,75^. The fit and predictability of the models were determined by *R*^2^Y and *Q*^2^Y values, respectively. A heatmap (Fig. 7a) visualizing quantifications of metabolites was generated by the ‘ComplexHeatmap’ package^76^ in R Studio (v.1.4.1106). PLSR was performed with luminal metabolites as predictors and blood glucose, insulin, GIP, GLP-1 and PYY as outcomes (Fig. 7b,d). PLSR was conducted using the ‘plsr’ package in R^77^. Data were scaled enabling each variable to contribute uniformly to the model. The number of principal components used was determined through the Root mean square error of prediction to achieve the highest prediction accuracy. The model was validated by the ‘leave one out’ method. Correlation networks (Fig. 7c,e) were created to highlight significant correlations between luminal metabolites and blood responses. The FDR-adjusted *P* values were calculated using the Benjamini–Hochberg method, with a significance threshold set at 0.05.

### Reporting summary

Further information on research design is available in the Nature Portfolio Reporting Summary linked to this article.

## Supplementary information

Supplementary Information Supplementary Texts 1–3; Tables 1–8; Fig. 1 and 2 and consort diagram.

Reporting Summary

Supplementary Data 1 Raw NMR data for Fig.6 and Extended Data Fig.6.

## Source data

Source Data Fig. 2 Statistical Source Data.

Source Data Fig. 3 Statistical Source Data.

Source Data Fig. 4 Unprocessed microscopic images.

Source Data Fig. 5 Statistical Source Data.

Source Data Fig. 7 Statistical Source Data.

Source Data Extended Data Fig. 2 Statistical Source Data.

Source Data Extended Data Fig. 3 Statistical Source Data.

Source Data Extended Data Fig. 4 Statistical Source Data.

Source Data Extended Data Fig. 5 Statistical Source Data.

Source Data Extended Data Fig. 7 Statistical Source Data.

Source Data Extended Data Fig. 8 Statistical Source Data.

## Data Availability

The data reported in this study are available from the Mendeley Data Database at https://data.mendeley.com/datasets/4vn35twm9v/1. The data related to the figure can be accessed through Figshare at 10.6084/m9.figshare.c.7838006.v1. The Human Metabolome Database (http://hmdb.ca/) and the Biological Magnetic Resonance Data Bank (https://bmrb.io/) were used for metabolite identification. Source data are provided with this paper.

## Code availability

This study does not report original code.
