# Supplementary material for: Upper-gastrointestinal tract metabolite profile regulates glycaemic and satiety responses to meals with contrasting structure: a pilot study
Source: Nat Metab. 2025 Jun 20;7(7):1459–75. doi: 10.1038/s42255-025-01309-7 (PMC12286859; doi:10.1038/s42255-025-01309-7)
Supplement: Supplementary file 1 — Supplementary Texts 1–3; Tables 1–8; Fig. 1 and 2 and consort diagram. [file 42255_2025_1309_MOESM1_ESM.pdf]

nature metabolism

Supplementary information https://doi.org/10.1038/s42255-025-01309-7

Upper-gastrointestinal tract metabolite profile regulates glycaemic and satiety responses to meals with contrasting structure: a pilot study

In the format provided by the authors and unedited

Supplementary Information

Table of Contents

Supplementary Text 1. Dietary intervention and meal preparation................................2

Supplementary Text 2. Study protocol 4

Supplementary Text 3. Serum profiling and metabolites quantification 14

Supplementary Table 1. Inclusion and exclusion criteria...15

Supplementary Table 2. Participant characteristics (n=10) 16

Supplementary Table 3. Overview of intestinal samples used for analyses[1] 17

Supplementary Table 4. Peak parameters of the glycemic, insulinemic and gut hormone responses to meal type [1] 18

Supplementary Table 5. Mean analyte concentrations in aspirated gastric and duodenal samples after meals with different structures...20

Supplementary Table 6. pH values of gastric aspirates..21

Supplementary Table 7. Metabolite identifications for NMR spectra...............................22

Supplementary Table 8. List of metabolites that are associated with the difference between food structure interventions in Partial Least Squares-Discriminant Analysis (PLS-DA) models (Fig. 6B-E) 24

Supplementary Table 9. Within-subject variability of blood glucose and hormone responses across intervention days 25

Supplementary Table 10. Paired Wilcoxon signed-rank tests for serum metabolites between food structure interventions...26

Supplemtary Figure 1. Visualization of algorithmically fitted pseudo-Voigt profiles used for metabolite quantification. 27

Supplemtary Figure 2. Validation of metabolite quantifications through correlation between separate signals 28

Supplementary Text 1. Dietary intervention and meal preparation

Dietary intervention: All test meals were prepared from the same batch of whole chickpeas, Cicer arietinum L., Kabuli type (Argentine variety, supplied by AGT Poortman Ltd.), which were abrasively dehulled, dry-milled (if applicable) and sieved, then weighed into test-meal specific portions, labelled, and sealed by a researcher independent from this study. Cooked test meals were prepared fresh for each study visit, using the concealed ingredient portions with the corresponding standardised cooking programme and a Vorwerk Thermomix Version 5 (see below for further details) which was developed to enable reproducible production of meals with contrasting structures. The portion size was controlled based on the moisture content of the chickpea portion to ensure delivery of 30 g total starch per serving for all meal types. Typically, the freshly cooked chickpea serving (containing 60 g chickpea dry solids) weighed 490, 280 and 230 g for meals Broken, Intact-S, and Intact-C, respectively, reflecting their different water content, and were served with 270, 480, and 530 g water to achieve a consistent total portion size of 760 g, including test meal, water and flavouring (15 g 'no sugar added blackcurrant jam', Stute Foods Ltd., Bristol, UK, and 115 g 'Hartley's no added-sugar raspberry flavoured jelly', Histon Sweet Spreads Ltd., Leeds, UK). Based on proximate analysis of the chickpea component (performed by accredited food testing provider ALS Laboratories Ltd., Chatteris, UK) and nutrition labels on food packaging (jelly and jam), each test meal serving provided (mean of triplicate with SD, 41.16 ± 0.4 g available carbohydrate of which 29.5 ± 0.04 g total starch and 2.29 ± 0.00 g sugars, 6.58 ± 0.43 g dietary fiber, 11.02 ± 0,03 g protein, 3.74 ± 0.06g fat for a total 242.7 ± 0.76 kcal. Thus, all meals contained the same ingredients and macronutrient composition per serving but were designed to differ in microstructure, i.e., consisting of either mainly broken cells (Broken), individual cells (Intact-S) or cell clusters (Intact-C).

Meal Preparation: Chickpeas were the main ingredient of the test meals. Whole chickpeas *Cicer arietinum* L., Kabuli type, Argentine variety (7mm diameter) were supplied by AGT Poortman Ltd. These were dehulled through an abrasive process by holding 200 g batches of dry chickpea (as supplied) in a Steelbrush dehuller (Westrup pre-cleaning debranner, coarse blue roll) for 30s, then sieved on a 3mm grid to exclude fragments of testa. The resulting dehulled chickpea material was weighed out into 75.0 g portions (by a researcher independent to the study), sealed and labelled. These dehulled chickpea portions were the main ingredient for the '*broken cell'* (Broken) and '*intact separated cell'* (Intact-S) chickpea meals, which were freshly prepared on the morning of each study visit using standardised processing protocols to deliver different structures. For the ***'****cell cluster'* (Intact-C) meal, the dehulled chickpeas were further processed by milling to obtain intact tissue particles (1.6-2.0 mm) containing whole cells, before portioning out. These coarse particles were obtained by trickle feeding the dehulled chickpeas through a Buhler miag Vario mill set up with a Roll Gap of 1.8 mm and Roller speed 220:450 rpm on 24.5 mm diameter break rolls 5 flutes/cm break rolls in Sharp-Sharp disposition for the first break. Output material was analytically sieved to collect target size fraction (≥ 1.6 and < 2.0 mm),

and the oversize material (≥ 2.0 mm) was then re-milled according to the same setting as used for the first break, but with a smaller Roll Gap (0.8 mm), to increase recovery of the target size fraction. This coarsely milled chickpea material (≥1.6 and <2.0 mm 'semolina') was the main ingredient for the 'cell-cluster' meal, and was weighed into 75 g portions in preparation for the study visits.
Each chickpea ingredient portion was then processed further, as described below, using a Thermomix® TM5 (Vorwerk) food processor to deliver freshly cooked meals with different structures to the study participants.

For the 'Broken cell' and 'Separated cells' meal types, the pre-weighed 75 g portion of de-hulled chickpeas was rinsed with drinking water from the tap in a colander until the water ran clear, and then immersed in 1L tap water under cover overnight for ~16 h. The following morning, the soaked chickpeas were then drained, rinsed briefly with fresh tap water, and drained thoroughly before transferring to the Thermomix. For the **Broken** meal, additional tap water was then added to the soaked chickpea to achieve a total weight of 637.5 g. This mixture was then blended (forward direction, speed setting 10) for 3 min at 37 °C, which breaks the chickpea cotyledon cells, before further heating and cooking at 95 °C for 28 min, with gentle stirring (blender in reverse setting, speed setting 4). A portion (~490 g) of the cooked mixture was then weighed into a bowl and seasoned with a 115 g pot of jelly (Hartleys' no added sugar raspberry jelly) and 15 g of sugar-free jam (Stutes no added sugar blackcurrant jam), to aid palatability, and served with drinking water (~270 mL) and served immediately to participants while still warm (above 60 °C).

The **Intact-S** meal, additional tap water was added after the overnight soak to achieve a total weight of 375.0 g, and then covered, heated and cooked at 95 °C for 80 min, with gentle stirring (blender in reverse direction, speed setting 5). A portion (~280 g) of the cooked mixture was then weighed into a bowl, seasoned as described above, and served immediately while still warm, together with drinking water (~480 mL).

For the **Intact-C** meal, the soaked semolina were drained and transferred into the Thermomix together with additional tap water to achieve a total wet weight of 300 g. This was then cooked for 50 min at 95 °C with gentle stirring (reverse, speed setting 1). A serving of ~120 g drained cooked chickpea solids was then transferred into a bowl, seasoned as above, and served immediately while warm together with drinking water.

For all meals, the volume of water served in the drinking glass was adjusted based on the cooked meal weight, so that all meals provided 30 g total starch from chickpea, the same amount of seasoning and similar total mass (~760 g).

Supplementary Text 2. Study protocol

1. INTRODUCTION

A number of reports globally demonstrate the rates of obesity and type 2 diabetes continue to increase. It has been estimated that more than 50% of the adult population are overweight (Wang, 2011) and that one in 17 people has either diagnosed or undiagnosed diabetes in the UK (Diabetes, U. K, 2014). These statistics highlight the importance of the maintenance of a energy balance and glucose homeostasis.

Chickpeas are an excellent source of high-quality protein and dietary fibre. Previous research has reported that chickpea starch is more resistant to digestion in the small intestine, which associates with lower bioavailability of glucose and improved bowel health (Nestel, 2004). It has been demonstrated that a controlled diet with chickpeas results in decreases in plasma glucose and insulin concentration (Nestel, 2004), as well as reductions in serum total cholesterol (TC) and low-density lipoprotein-cholesterol (LDL-C) (Pittaway, 2007), potentially contributing to lower risk of type 2 diabetes and coronary heart disease (CHD). Furthermore, compared to a wheat-based meal, greater satiety was reported by some participants (Pittaway, 2007; Zafar, 2017), possibly leading to lower energy intake and improved weight control. However, there has been limited research into chickpea and its potential health benefits and the stimulation of gut hormone secretion. Chickpeas are high in resistant starches and protein, and these nutrients have been shown to stimulate gut hormone secretion, including gastric-inhibitory peptide (GIP), glucagon-like peptide-1 (GLP-1) and peptide YY (PYY) that could regulate glucose homeostasis (Raben, 1994; Zhou, 2008; Smeets, 2008). These suggest that further research is needed to investigate the relationship between chickpeas supplementation and gut hormone secretion.

The microstructure of plant food, which is altered during processing such as grinding, heating or fermentation that could break cellular structure and thereby influence Type 1 Resistant Starch content, has been shown to affect nutrient bioavailability and digestion process (Sensory, 2014). Thus, this project will improve understanding of the relationship among food structure ranging in processing, nutrient bioavailability and chickpea-induced release of PYY, GIP and GLP-1.
The present study will combine two different methodologies for sampling from the gastrointestinal tract, which have been used in previous studies by our research group (REC references: 17/LO/0354, 15/LO/0184).

Overall, the aim of this study is to investigate the impact of different chickpea tissue-structures on gut hormone secretion, thus explaining the chickpeas' influences on glucose control and satiety reported in previous studies.

2. STUDY METHODOLOGY

Participants: 15 healthy male and female volunteers. This is a pilot study in a new area and therefore a formal power calculation is not possible.

Recruitment: Participants will be recruited from existing healthy volunteer databases and by advertisement in public places. Adverts will be placed in newspapers and put up in public buildings. A contact number on the advert will enable potential participants to contact the research team at Imperial College London. Participation in the study will be entirely voluntary. No undue influence will be exerted by the researchers. Participants will be free to withdraw from the study at any time.

Once potential participants have responded to a study advertisement a researcher will arrange a short telephone interview to explain the study. Following this a more formal interview will be arranged in order for the researcher to determine if the potential participant meets the inclusion and exclusion criteria. This also gives the potential participants a chance to ask any questions they may have about the study. Prior to the interview they will have been sent a written participant information sheet by email or by post.

2.1. STUDY DESIGN

This will be a randomised cross-over feeding study where volunteers will be clinical research facility inpatients for four days on study visit 1 and 3 days on study visit 2,3,4.There will be at least 3 days between Study Visit 1 and 2. There will be at least 7 days between visits 2-3 and 3-4.
Health Screening:

Participants will attend the NIHR/Wellcome Trust Imperial Clinical Research Facility at Hammersmith Hospital where their eligibility will be assessed. A pre-screening questionnaire will be done to record the personal information, body composition and medical history.They will have a blood test (HbA1c, FBC, LFT, U&E and lipids) and height and weight measurements will also be taken. They will also have an electrocardiogram (ECG) and blood pressure will be recorded. All women of child bearing age will have a pregnancy test.

Study Visit 1

The day prior to the study visit, the participants will be requested to refrain from strenuous exercise and alcohol and to arrive having fasted from the evening before. The following morning participants will be asked to attend the NIHR Imperial Clinical Research Facility (CRF) at Hammersmith Hospital for 4 days (3 nights).

Day 1

Enteral feeding tubes will be placed to allow for sampling of intestinal content from the stomach and small intestine throughout the study visit. The enteral feeding tubes will be placed by a trained

medical professional in the Imperial Clinical Research Facility using the CORPAK feeding tubes that track the position of the tube during placement without the need for x-rays. This system has been used in previous studies by our research group (REC Ref: 15/LO/0184). These tubes will remain in place for the duration of the 4 day visit. The diet of all participants will be standardised throughout the visit.

Day 2-4

On the morning of Day 2, an intravenous cannula will be inserted to allow for blood sampling and will remain in place for the remainder of the study visit.

Each morning, two fasting blood samples and gastric and duodenal samples will be taken prior to the test meal. In addition, two baseline subjective appetite measures, assessed by visual analogue scale, and two baseline breath hydrogen measurements will be collected in real-time using a gastrolyser.

Each day, in a random order, participants will then receive a standardised breakfast made from cooked chickpeas with different structures:
1. Broken cells from chickpeas (control)
2. Individual cells from chickpeas
3. Clusters of cells from chickpeas

The control breakfast will provide the same total starch content as the test interventions. The chickpea material will be provided by Quadram Institute Bioscience.The recipe of the breakfast will be designed by the research group from Imperial College London and Quadram Institute Bioscience. The food material will be purchased from Sainsbury's or another UK food supermarket. Volunteers will be randomized using the 'sealed envelope' website.

Further blood samples will be collected at 15, 30, 45, 60, 90, 120, 150, 180 min following the test meal in order to measure hormones, metabolites and inflammatory signals. 10 ml of blood will be taken at each blood sample. 300 ml of blood will be taken during the 4 day study visit.

Gastric and duodenal content will be taken at 15, 30, 45, 60, 75, 90, 105, 120, 135, 150, 165, 180 min. Microscopy and metabolomics assessment will be performed to assess the impact of initial digestion on the breakdown of the different chickpea structures.

Subjective appetite measures will be collected at 0, 30, 60, 90, 120, 150, 180 min using VAS questionnaire. Breath hydrogen will be measured at 0, 60, 120, 180 min.

Urine will be collected throughout the study visit to measure metabolite concentrations. Subjects will empty their bladder before the test breakfast and collect all urine thereafter for a period of 180 min. Subjects will be provided with an appropriate measurement container to collect urine.

Following the 180 min sample on the fourth study day, the enteral tubes and intravenous cannula

will be removed and volunteers will be discharged from the Clinical Research Facility.

A lunchtime meal (at 4 h) will be provided in surplus, and dietary intakes recorded such that the participant's ad libitum energy intake can be determined. A fixed dinner-time meal will be provided at 9 h. Water (for the first 4 h) and beverages are provided throughout the day.

After study visits, gastric and duodenal samples will be sent to Quadram Institute Bioscience and stored for subsequent analysis.

Study Visits 2,3,4

Study Visits 2,3, and 4 are focussed on events occurring in the lower small intestine, and the microbial and metabolic responses to these events.

Day 1

The day prior to each 3-day study visit, participants will be requested to refrain from strenuous exercise, caffeine and alcohol. Participants will then be requested to fast overnight (they are allowed to drink water) and will come to the Imaging Department at Charing Cross Hospital for the insertion of nasoenteric tubes. Following the tube insertion, participants will return to the NIHR Imperial CRF to complete the three day study. They will return to the CRF at Hammersmith Hospital in a taxi and will be accompanied by a member of the study team.

On day 1 of the study, all female participants of child bearing age will be asked to provide a urine specimen in order to perform a pregnancy test prior to placement of the tube. A nasoenteric tube will then be inserted through the nose, with a small balloon at the terminal end which is inflated and used to carry the tube through the small intestine by peristalsis. Once the tube reaches the terminal ileum, the balloon is deflated and the tube is restrained from additional movement for the rest of the 3-day study visit. The tube position will be confirmed by fluoroscopy and administration of diluted barium sulphate. The tubes will be inserted at Charing Cross Hospital following a procedure which has been used in previous studies by our research group (17/LO/0354). Following the tube insertion, participants will travel in a taxi accompanied by a member of the study team to the NIHR/Wellcome Trust Imperial CRF to complete the four day study visit.

During these 3 separate study visits, volunteers will be provided with chickpea-enriched diets differing in which the structure of the chickpea component differs. The chickpea material will be provided by Quadram Institute Bioscience. The recipe of the diet will be designed by the research group from Imperial College London and Quadram Institute Bioscience. The food material will be purchased from Sainsbury's or another UK food supermarket Each visit, in a randomised order, volunteers will receive a standardised low-fibre background diet supplemented with one of the following:

1. broken cells from chickpeas (control)
2. individual cells from chickpeas
3. chickpea cell clusters

Volunteers will be fed one of the diets over the 3-day study period. The diet will start following the tube placement on Day 1 and end on Day 3. Volunteers will also be asked to collect a stool sample on each day of the 3-day study period.
Volunteers will be randomized using the 'sealed envelope' website.
Ileal sample collection will start <u>on day 2</u>, with two baseline samples taken before breakfast and 60, 120, 180, 240, 300, 360, 420 and 480 min after breakfast for metabolomic analysis, which will include 1H NMR spectroscopy, ultra-performance LC-MS and GC-MS, microbiological analysis and for microscopy.
<u>On day 3</u>, ileal samples will be collected as described on day 2 and will be matched with blood sampling and visual analogue scales (VAS) to assess appetite responses. An intravenous cannula will be inserted on the morning of day 3 and two fasting blood samples will be collected before breakfast. After breakfast, blood samples will be collected at 60, 120, 180, 240, 300, 360, 420 and 480 min to measure hormones and metabolites. 100 ml (10 × 10 ml) will be collected throughout each study visit. At each time point, volunteers will be asked to complete VAS to assess subjective feelings of hunger, fullness and nausea. Breath hydrogen concentrations will also be measured at the same time.
At the end of day 3, the cannula and the nasoenteric tube will be removed. It is expected that the majority of participants will have the naso-enteric tubes removed through the nose at the CRF Unit. If more than mild discomfort seems likely to be caused by the removal of the tube, they will be transported to Charing Cross Hospital in a taxi accompanied by a trained medical professional and will have the tube removed under fluoroscopy, or the nasal end of the tube will be cut and the rest of the tube will be allowed to pass rectally.
Collected tissue samples stored may be used in future ethically approved studies.
After study visits, ileum and stool samples will be sent to Quadram Institute Bioscience and stored for subsequent analysis.

2.2. INCLUSION/ EXCLUSION CRITERIA

<u>Inclusion criteria:</u>

- Male or female
- Age between 18-65 years (inclusive)
- Body mass index (BMI) of 18-30 kg/m2
- Willingness and ability to give written informed consent and willingness and ability to understand, to participate and to comply with the study requirements

<u>Exclusion criteria:</u>

- Abnormal ECG
- Screening blood results outside of normal reference values
- Weight change of ≥ 5kg in the preceding 2 months
- Current smokers

• History of substance abuse and/or excess alcohol intake • Pregnancy • Diabetes • Cardiovascular disease

• Cancer

• Gastrointestinal disease e.g. inflammatory bowel disease or irritable bowel syndrome

• Kidney disease

• Liver disease

• Pancreatitis

• Started new medication within the last 3 months likely to interfere with energy metabolism, appetite regulation and hormonal balance, including: anti-inflammatory drugs or steroids, antibiotics, androgens, phenytoin, erythromycin or thyroid hormones.

• Participation in a research study in the 12 week period prior to entering this study.

• Any blood donation within the 12 week period prior to entering this study

Any participants with the above conditions would already have an altered pattern of hormones and inflammatory molecules because of their disease process and would therefore give us confounding or misleading results.

Support of number of volunteers: This is a pilot study in a new area and therefore a power calculation is not possible.

3. WITHDRAWL CRITERIA AND ADVERSE EVENTS

3.1. WITHDRAWAL CRITERIA

The safety of the study participants takes priority. Any significant adverse event (as assessed by the researchers) will halt the study and the ethics committee and sponsor will be informed as per standard protocol. All adverse events will be recorded and investigators will review each adverse event as it arises. In addition, participants will be free to withdraw at any time and are not required to give a reason.

3.2. ADVERSE EVENTS

Adverse Event (AE): Any untoward medical occurrence in a patient or clinical study subject.

Serious Adverse Event (SAE): Any untoward and unexpected medical occurrence that:

- results in death
- is life- threatening – refers to an event in which the subject was at risk of death at the time of the event; it does not refer to an event which hypothetically might have caused death if it was more severe.
- requires hospitalisation, or prolongation of existing inpatients’ hospitalisation.
- results in persistent or significant disability or incapacity
- is a congenital abnormality or birth defect

Medical judgement should be exercised in deciding whether an AE is serious in other situations. Important AEs that are not immediately life threatening or do not result in death or hospitalisation but may jeopardise the subject or may require intervention to prevent one of the other outcomes listed in the definition above, should also be considered serious.

4. REPORTING PROCEDURES

All adverse events should be reported. Depending on the nature of the event the reporting procedures below should be followed. Any questions concerning adverse event reporting should be directed to the Chief Investigator in the first instance.

4.1. Non-serious AEs

All such events, whether expected or not, should be recorded.

4.2. Serious AEs (SEAs)

An SAE form should be completed and emailed to the Chief Investigator within 24 h. However, relapse, death and hospitalisations for elective treatment of a pre-existing condition do not need reporting as SAEs.

All SAEs should be reported to the XXXX Research Ethics Committee where in the opinion of the Chief Investigator the event was:

- ‘related’, i.e. resulted from the administration of any of the research procedures; and
- ‘unexpected’, i.e. an event that is not listed in the protocol as an expected occurrence.

Reports of related and unexpected SAEs should be submitted within 15 days of the Chief Investigator becoming aware of the event, using the NRES SAE form.

Local investigators should report any SAEs to the sponsor and their Local Research Ethics Committee and/ or Research and Development Office.

Contact details for reporting SAEs
Email g.frost@imperial.ac.uk, attention Professor Gary Frost
Please send SAE forms to Professor Gary Frost
Tel: 020 838 33242 (Mon to Fri 09.00- 17.00)

5. REGULATORY ISSUES

5.1. ETHICS APPROVAL

The Chief Investigator has obtained approval from the HRA and Research Ethics Committee. The study must also receive confirmation of capacity and capability from each participating NHS Trust

before accepting participants into the study. The study will be conducted in accordance with the recommendations for physicians involved in research on human subjects adopted by the 18th World Medical Assembly, Helsinki 1964 and later revisions

5.2. CONFIDENTIALITY

The Chief Investigator will preserve the confidentiality of participants in the study and is registered under the Data Protection Act. The Principal Investigator will preserve the confidentiality of participants taking part in the study and is registered under the Data Protection Act 2018. Signed consent forms will be kept in a locked filing cabinet in a locked office in the Section of Investigative Medicine, Imperial College London. These forms will contain the participant names and an individual study code. All other data will contain the individual study code and no other participant identifying information. This will make data anonymous.

Anonymised data will securely forward to researchers in the Food Innovation Health Programme at Quadram Institute Bioscience,located at Norwich Research Park, Colney Ln, Norwich NR4 7UA, after a non-disclosure agreement specific to this study is signed

5.3. INDEMNITY

Imperial College holds negligent harm and non-negligent harm insurance policies, which apply to this study.

5.4. SPONSOR

Imperial College London will act as the main sponsor for this study. Delegated responsibilities will be assigned to the NHS trusts taking part in this study.

5.5. FUNDING

The study is funded by a BBSRC Strategic Programme Grant (BB/R012512/1) awarded to the Quadram Institute Bioscience (QIB). Prof. Gary Frost is a Principal Investigator within the QIB Food Innovation and Health Programme (lead PI: Prof. Richard Mithen) and has been allocated funds (£688,179) for completion of this study.

5.6. REIMBURSMENT AND CONSENT

Recruitment posters will be placed on Imperial College London campuses and Imperial College Healthcare NHS Trust Sites (South Kensington, Hammersmith Hospital, Charing Cross,St. Mary’s and Queen Charlotte’s Hospital). Adverts may also be placed in newspapers or magazines, or on the Imperial College Healthcare NHS Trust Internet Homepage and will use the same text as that used on the recruitment poster, although layout may vary.

Written and informed consent will be taken by a member of the research team who has experience in obtaining informed consent. Those participants who agree to take part will be asked to sign a consent form before any study procedure is started. Participation in the study will be entirely

voluntary. No undue influence will be exerted by the researchers. Participants will be free to withdraw from the study at any time.

Participants will not be paid for taking part in this study to avoid any possible feelings of coercion. However, in recompense for travel expenses, loss of earnings and the significant burden of repeated trips to the hospital, £10 for the screening visit and £500 for study visit 1, £350 for study visit 2,3,4 respectively will be made available for participants. This amount also reflects similar expense payments offered in previous studies by our research group.

5.7. AUDITS AND INSPECTIONS

The study may be subject to inspection and audit by Imperial College London under their remit as sponsor and other regulatory bodies to ensure adherence to GCP and The UK Policy Frame Work for Health and Social Care Research

6. PUBLICATION POLICY

The findings of the research will be published in an open-access, peer-reviewed journal. In addition we will be collaborating with patient groups and professional groups to disseminate the findings via multiple media channels such as patient association publications, print and broadcast media. No participants identifiable data will be included.

7. REFERENCES

Diabetes, U. K. (2014). Diabetes: facts and stats. *Diabetes UK*, *3*, 1-21.Nestel, P., Cehun, M., & Chronopoulos, A. (2004). Effects of long-term consumption and single meals of chickpeas on plasma glucose, insulin, and triacylglycerol concentrations. *The American journal of clinical nutrition*, *79*(3), 390-395.

Nestel, P., Cehun, M., & Chronopoulos, A. (2004). Effects of long-term consumption and single meals of chickpeas on plasma glucose, insulin, and triacylglycerol concentrations. *The American journal of clinical nutrition*, *79*(3), 390-395.

Pittaway, J. K., Ahuja, K. D., Robertson, I. K., & Ball, M. J. (2007). Effects of a controlled diet supplemented with chickpeas on serum lipids, glucose tolerance, satiety and bowel function. *Journal of the American College of Nutrition*, *26*(4), 334-340.

Raben, A., Tagliabue, A., Christensen, N. J., Madsen, J., Holst, J. J., & Astrup, A. (1994). Resistant starch: the effect on postprandial glycemia, hormonal response, and satiety. *The American journal of clinical nutrition*, *60*(4), 544-551.

Sensoy, I. (2014). A review on the relationship between food structure, processing, and bioavailability. *Critical reviews in food science and nutrition*, *54*(7), 902-909.

Smeets, A. J., Soenen, S., Luscombe-Marsh, N. D., Ueland, & Westerterp-Plantenga, M. S. (2008). Energy expenditure, satiety, and plasma ghrelin, glucagon-like peptide 1, and peptide tyrosine-tyrosine concentrations following a single high-protein lunch. *The Journal of nutrition*, *138*(4), 698-702.

Wang, Y. C., McPherson, K., Marsh, T., Gortmaker, S. L., & Brown, M. (2011). Health and economic burden of the projected obesity trends in the USA and the UK. *The Lancet, 378(9793), 815-825.*

Zafar, T. A., & Kabir, Y. (2017). Chickpeas suppress postprandial blood glucose concentration, and appetite and reduce energy intake at the next meal. *Journal of food science and technology*, *54*(4), 987-994.

Zhou, J., Martin, R. J., Tulley, R. T., Raggio, A. M., McCutcheon, K. L., Shen, L., ... & Keenan, M. J. (2008). Dietary resistant starch upregulates total GLP-1 and PYY in a sustained day-long manner through fermentation in rodents. *American Journal of Physiology-Endocrinology and Metabolism*, *295*(5), E1160-E1166.

Supplementary Text 3. Serum profiling and metabolites quantification

Serum samples were thawed and then centrifuged for 10 min at 3,000g at 4 °C. 350 µL of serum mixed with 350 µL of phosphate buffer (75 mM Na2HPO4, 2 mM NaN3, and 4.6 mM sodium trimethylsilyl propionate-[2,2,3,3-2H4] (TSP) in D2O, pH 7.4 ± 0.1). 600ul µL of the mixture were transfer to the 5mm NMR tubes. NMR spectroscopic analyses were performed on a 600 MHz Bruker Avance III HD spectrometer equipped with a 5mm BBI probe and fitted with the Bruker SampleJetTM robot cooling system set to 5°C. A full quantitative calibration was completed prior to the analysis using a previously described protocol [71]. A total of two experiments were completed in automation mode: a standard 1D experiment with solvent presaturation (32 scans, 96 K data points, and a spectral width of 30 ppm), a Carr–Purcell–Meiboom–Gill (CPMG) spin-echo experiment (32 scans, 72 K data points, and a spectral width of 20 ppm) which filters the spectrum by differential T2 relaxation removing the peaks from the large molecules. Absolute quantifications from 1H-NMR spectra were performed with Bruker IVDr software: B.I.Quant-PS 2.0.0 to quantify 41 serum metabolites (mmol/L units). However, for the purpose of this paper we focused on 11 serum metabolites that were also quantified at gastric and duodenal samples.

Supplementary Table 1. Inclusion and exclusion criteria

Inclusion Criteria	Exclusion Criteria
Male or female	Abnormal ECG
Age between 18-65 years (inclusive)	Screening blood results outside of normal reference values.<br>Fasting glucose 3.9-5.6 mmol/L<br>Haemoglobin M125-170/ F114-150 g/L<br>White blood cell count 4.2-11.2 x 10^9/L<br>Red blood cell count 3.73-4.96 million/mcL
Body mass index (BMI) of 18-30 kg/m^2	Weight change of ≥ 5kg in the preceding 2 months
Willingness and ability to give written informed consent and willingness and ability to understand, to participate and to comply with the study requirements	Current Smokers
	History of substance abuse and/or excess alcohol intake<br>Cancer<br>Gastrointestinal disease e.g., inflammatory bowel disease or irritable bowel syndrome, kidney disease, liver disease and pancreatitis<br>Pregnancy<br>Diabetes or cardiovascular disease
	Participant in a research study or blood donation 12 weeks prior<br>Started new medication within the last 3 months likely to interfere with glucose and energy metabolism, appetite regulation and hormonal balance, including: anti-inflammatory drugs or steroids, antibiotics, androgens, phenytoin, erythromycin, or thyroid hormones.

Supplementary Table 2. Participant characteristics (n=10)

Variable	Baseline measurements
Gender (M:F)	6:4
Age (years)	30.8 ± 2.41[1]
Height (m)	1.70 ± 0.02[1]
Weight (kg)	72.07 ± 3.20[1]
BMI (kg/m^2)	24.91 ± 0.82[1]
Body Fat (%)	17.35 ± 1.91[1]

[1]Results presented as Mean ± SEM

Supplementary Table 3. Overview of intestinal samples used for analyses[1]

	Gastric Supernatants			Gastric Pellets			Duodenal Supernatants			Duodenal Pellets		
Time (min)	**Intact-S**	**Intact-C**	**Broken**	**Intact-S**	**Intact-C**	**Broken**	**Intact-S**	**Intact-C**	**Broken**	**Intact-S**	**Intact-C**	**Broken**
-10	7	4	3	3	4	3	3	2	1	0	0	0
0	6	4	3	5	3	3	2	3	2	0	0	0
15	8	8	9	8	6	9	6	6	4	3	1	2
30	9	9	9	9	9	9	7	7	3	5	2	1
45	9	7	9	8	7	9	6	6	8	2	3	5
60	9	9	9	8	9	9	4	7	6	3	3	4
75	9	8	9	9	7	9	9	5	7	5	3	5
90	9	9	9	9	9	9	4	2	6	3	1	5
105	9	8	9	8	7	9	3	4	4	4	0	3
120	9	5	8	9	3	8	4	7	6	4	2	2
135	9	6	8	9	6	8	4	6	7	2	0	4
150	9	7	7	9	6	7	5	4	5	2	2	4
165	7	7	6	7	5	6	4	4	3	4	1	2
180	8	5	8	7	5	6	4	2	3	5	0	1

[1] The reason for lower numbers of samples used in analyses was that the volume of sample collected was not always sufficient for further analyses to be conducted.

Supplementary Table 4. Peak parameters of the glycemic, insulinemic and gut hormone responses to meal type [1]

	Intact-S	Intact-C	Broken
**Plasma Glucose**	Time x Treatment *P* < 0.001		
Fasting (mM)	4.94 (4.57 5.36)	4.98(4.63 5.37)	5.09 (4.80 5.42)
iPeak (mM)	**0.76 (0.43 1.34)^{a}**	**0.65(0.22 0.97)^{a}**	**1.80(1.36 2.30)^{b}**
Peak X (min)	41(23 74)	26(18 39)	35(25 42)
Last X (min)	87(56 136)	62(33 117)	105(81 136)
iAUC (mM.min^{-1})	**31.4(11.5 85.9) ^{ab}**	**12.7(2.9 54.9)^{a}**	**81.0(55.1 119.2) ^{b}**
**Plasma Insulin**	Time x Treatment *P* = 0.006		
Fasting (µU/mL)	6.95(5.43 9.28)	8.91(7.88 10.26)	8.15(6.57 10.74)
iPeak (µU/mL)	**22.0(15.4 31.3)^{ab}**	**13.5(9.3 19.4)^{a}**	**36.2(23.4 55.9)^{b}**
Peak X(min)	39(23 66)	25(18 34)	37(23 56)
Last X (min)	**163(135 196) ^{a}**	**74(46 120) ^{b}**	**136(116 162) ^{a}**
iAUC (µU/mL.min^{-1})	**1538(986 2398)^{a}**	**410(235 715)^{b}**	**1910(1384 2636)^{a}**
**Plasma GIP**	Time x Treatment *P* =0.006		
Fasting (pg/mL)	47.29(38.88 59.62)	51.58(42.48 64.90)	51.90(44.06 62.78)
iPeak (pg/mL)	**66.8(54.2 82.2) ^{a}**	**26.7(15.0 47.4) ^{b}**	**84.3(68.0 104.6) ^{a}**
Peak X (min)	**113(77 166) ^{a}**	**88(43 180) ^{a}**	**39(25 49) ^{b}**
Last X (min)	180(180,180)	151(112 204)	160(132 185)
iAUC (pg/mL.min^{-1})	**7267(4988 10587) ^{a}**	**1962(794 4847) ^{b}**	**6187(4383 8732) ^{a}**
**Plasma GLP-1**	Time x Treatment *P* =0.034		
Fasting (pM)	12.49(9.21 19.62)	12.51(9.54 17.44)	12.37(9.37 18.22)

iPeak (pM)	21.0(17.3 25.5)	11.4(6.2 21.1)	19.6(13.6 28.2)
Peak X (min)	**97(56 169)** [a]	**51(23 113)** [ab]	**29(20 43)** [b]
Last X (min)	180 (180, 180)	102(58 180)	123(80 190)
iAUC ($pM.min^{-1}$)	**2015(1668 2435)**[a]	**591(225 1555)**[ab]	**978(488 1960)**[b]
**Plasma PYY**	Log-transformed data: Time x Treatment *P* = 0.186, Treatment P=0.0158		
Fasting (pM)	11.12(7.53 17.28)	11.14(7.51 18.62)	10.62(7.59 17.13)
iPeak (pM)	**19.7(10.3 37.8)** [a]	**16.0(10.6 24.0)** [ab]	**7.4(5.0 10.9)** [b]
Peak X (min)	65(34 125)	35(18 72)	33(17 63)
Last X (min)	**120(69 206)**[a]	**66(33 129)**[ab]	**50(29 85)**[b]
iAUC ($pM.min^{-1}$)	**1049(350 3134)** [a]	**425(176 1028)** [ab]	**162(70 372)** [b]

[1] The fasting, the maximal postprandial rise (iPeak), time to peak (Peak X), time to return to baseline levels (Last X), and iAUC of the 1st peak values for blood glucose, plasma insulin, plasma GIP, GLP-1 and plasma PYY following consumption to macronutrient matched chickpea meals (30 g starch/serving) in which cotyledon cells are present as separated cells 'Intact-S', cell clusters 'Intact-C' or no longer cellular 'Broken'. Values were obtained from the postprandial time-series data, for which significance (P<0.05) of time x treatment interactions was determined by mixed-effects ANOVA. Data presented as geometric means with 95%CI. Superscript letters refer to pairwise comparisons' results (Tukey's post-hoc analysis); different letters represent significant differences at p < 0.05 probability level.

Supplementary Table 5. Mean analyte concentrations in aspirated gastric and duodenal samples after meals with different structures

Site	Fraction	Analyte	Intact-S	Intact-C	Broken	*P*-value
Gastric	Fluid	Maltose (mg/mL)	0.912 ± 0.465	0.6467 ± 0.171	3.04 ± 1.453	0.006
	Fluid	Sucrose (mg/mL)	0.192 ± 0.088	0.112 ± 0.043	0.233 ± 0.100	0.055
	Solids	Dry Matter (mg/mL)	39.04 ± 8.58	9.16 ± 1.95	15.44 ± 3.16	<0.001
Duodenal	Fluid	Maltose (mg/mL)	0.7539 ± 0.262	0.4907 ± 0.187	1.1800 ± 0.422	0.020
	Fluid	Maltotriose (mg/mL)	0.3632 ± 0.183	0.1229 ± 0.082	0.6265 ± 0.350	0.018
	Fluid	Glucose	0.3754 ± 0.175	0.1915 ± 0.096	0.4448 ± 0.153	0.003
	Fluid	Sucrose (mg/mL)	0.1115 ± 0.04715	0.0890 ± 0.049	0.1689 ± 0.058	0.041
	Solids	Dry Matter (mg/mL)	16.290 ± 1.260	9.840 ± 4.290	6.000 ± 2.778	0.009
	Solids	Total CHO (mg/mg DM)	0.536 ± 0.054	0.524 ± 0.042	0.427 ± 0.045	0.060
	Solids	Starch (mg/mg total CHO)	0.71 ± 0.028	0.682 ± 0.046	0.386 ± 0.065	<0.001
	Solids	Starch (mg/mL aspirate)	6.353 ± 2.678	3.263 ± 1.667	1.306 ± 0.456	<0.002

Values are means with 95% CIs obtained using samples collected from 10 participants up to 3h after test meal consumption. P-values are main effects by One Way RM ANOVA. Total CHO – Total carbohydrate is the total amount of sugars in acid hydrolysate of aspirated solids and is mainly from plant cell wall polysaccharides and starch

Supplementary Table 6. pH values of gastric aspirates

Subject	Diet	Time (min)			
		0	15	30	60
Subject 1	Intact-S	1.5	4	4	2.5
	Intact-C	1.5	4.5	4	2
	Broken	2	4.5	4	2
Subject 2	Intact-S	1	4	4	2
	Intact-C	1.5	4.5	4.5	2
	Broken	1	4	4	2
Subject 3	Intact-S	1.5	3.5	2	1
	Intact-C	1.5	3.5	2	1
	Broken	1.5	3.5	2	1.5
Subject 4	Intact-S	1.5	3.5	3.5	2
	Intact-C	1.5	4	4	2
	Broken	1	4	4	2
Subject 5	Intact-S	1.5	4	4	3
	Intact-C	1.5	4	4	2.5
	Broken	1.5	4	3.5	3

pH of gastric aspirates was measured by pH test strips.

Supplementary Table 7. Metabolite identifications for NMR spectra

Number[a]	Metabolite	Chemical shift (multiplicity)
1	Bile acids/lipids	0.67(s), 0.73(s), 0.92(s), 0.98(d), 2.26(m)
2	Valine	1.04(d)
3	Leucine/Isoleucine	0.94-0.97
4	Propionate	1.07(t)
5	Buyurate	1.55(q), 0.89(t)
6	Lactate	1.32(d),4.1(dd)
7	Alanine	1.48(d)
8	Acetate	1.92(s)
9	Methionine	2.143(s)
10	Glutamine	2.46(m)
11	Succinate	2.41(s)
12	Citrate	2.55(d),2.67(d)
13	Aspartate	2.82(dd)
14	Asparagine	2.962(dd)
15	Taurine-conjugated bile acids	3.08(t), 7.98-8.0(m)
16	Glycine-conjugated bile acids	offset at 7.91
17	Glucose	4.65(d), 5.24(d)
18	Maltose/Maltotriose	5.39-5.42
19	Oligosaccharides,(1→4)	5.34(d),5.36(d),5.374 (d), 5.382 (d))
20	Oligosaccharides (1→6)	4.96(d)-5.01 (m)
21	Stachyose/Raffinose	5.43 (d), 4.23 (d), 4.99 (d), 5.00 (d)
22	Fumarate	6.523(s)
23	Tyrosine	6.90(d), 7.20(d)
24	Histidine	7.10 (d), 3.16(dd), 3.23(dd)
25	Phenylalanine	7.34(m),7.38(m), 7.43(m)
26	Tryptophan	7.54(d), 7.74(d)
27	Formate	8.46(s)
28	Trigonelline	9.127(s),8,84(m),8.08(m),4.44(s)

[a]Number is related to the labels in **Figure 6A**. Multiplicity key is as follows: d: doublet, 2dd: doublet of doublet, 2xd: Two doublets s: singlet, t = triplet, m = (other) multiplet

Supplementary Table 8. List of metabolites that are associated with the difference between food structure interventions in Partial Least Squares-Discriminant Analysis (PLS-DA) models (Fig. 6B-E)

Models	Metabolites	Association	Adjusted- P values
**G30**	Tyrosine	↓	6.6E-07
	Phenylalanine	↓	5.8E-03
	Tryptophan	↓	3.1E-03
	Glucose	↑	7.4E-06
	Maltose	↑	1.2E-05
	Oligosaccharides,(1→4)	↑	1.3E-13
	Oligosaccharides (1→6)	↑	9.8E-09
**G120**	Glucose	↑	6.4E-10
	Maltose	↑	6.9E-03
	Oligosaccharides,(1→4)	↑	1.3E-03
	Oligosaccharides (1→6)	↑	9.8E-09
**D30**	total BAs	↓	1.2E-03
	TCBAs	↓	1.7E-02
	GCBAs	↓	1.1E-08
	Glucose	↑	1.1E-16
	Maltose	↑	1.4E-21
	Oligosaccharides,(1→4)	↑	3.2E-16
	Oligosaccharides (1→6)	↑	3.8E-09
**D120**	total BAs	↓	5.9E-04
	TCBAs	↓	4.4E-05
	GCBAs	↓	5.0E-05
	Valine	↓	5.4E-06
	Leucine/Isoleucine	↓	9.0E-05
	Alanine	↓	2.3E-08
	Formate	↑	1.9E-05
	Citrate	↑	8.1E-04

↑ indicates higher after ‘Broken’ meal, ↓ indicates higher after ’Intact’ meal. P-values were adjusted for multiple testing using the Benjamini-Hochberg False Discovery Rate (FDR). Abbreviations: G30, Gastric T=30min; G120, Gastric T=120min; D30, Duodenal T=30min; D120, Duodenal T=120min. BAs, bile acids; TCBAs, taurine-conjugated bile acids; GCBAs, glycine-conjugated bile acids.

Supplementary Table 9. Within-subject variability of blood glucose and hormone responses across intervention days

Fasting levels	Mean CV%
Blood glucose (mmol/L)	4.90
Blood GIP (pg/ml)	11.34
Blood GLP-1 (pmol/L)	16.90
Blood PYY (pmol/L)	24.61

CV, Coefficient of variation calculated based on repeated measures of the same individuals across all intervention days.

Supplementary Table 10. Paired Wilcoxon signed-rank tests for serum metabolites between food structure interventions

Serum	Broken vs Intact-C T30			Broken vs Intact-S T30			Intact-S vs Intact-C T30		
metabolites	P	P.adj	Sig.	P	P.adj	Sig.	P	P.adj	Sig.
Alanine	0.695	0.922	ns	0.232	0.696	ns	0.922	0.922	ns
Glutamine	0.386	0.407	ns	0.232	0.407	ns	0.407	0.407	ns
Methionine	0.155	0.193	ns	0.193	0.193	ns	0.074	0.193	ns
Asparagine	0.361	0.361	ns	0.272	0.361	ns	0.268	0.361	ns
Tyrosine	0.878	0.919	ns	0.683	0.919	ns	0.919	0.919	ns
Phenylalanine	0.183	0.549	ns	0.375	0.562	ns	0.906	0.906	ns
Histidine	0.539	0.808	ns	1	1	ns	0.375	0.808	ns
Valine	0.475	0.712	ns	1	1	ns	0.308	0.712	ns
Glucose	0.01	**0.029**	↑	0.02	**0.029**	↑	0.557	0.557	ns
Acetic acid	0.16	0.426	ns	0.475	0.475	ns	0.284	0.426	ns
Formic acid	0.26	0.39	ns	0.953	0.953	ns	0.16	0.39	ns

Serum	Broken vs Intact-C T120			Broken vs Intact-S T120			Intact-S vs Intact-C T120		
metabolites	P	P.adj	Sig.	P	P.adj	Sig.	P	P.adj	Sig.
Alanine	0.193	0.29	ns	0.922	0.922	ns	0.105	0.29	ns
Glutamine	0.432	0.432	ns	0.407	0.432	ns	0.432	0.432	ns
Methionine	0.139	0.209	ns	**0.032**	0.097	ns	0.262	0.262	ns
Asparagine	0.584	0.584	ns	0.076	0.228	ns	0.294	0.441	ns
Tyrosine	0.058	0.173	ns	0.307	0.46	ns	0.722	0.722	ns
Phenylalanine	0.507	0.906	ns	0.813	0.906	ns	0.906	0.906	ns
Histidine	0.322	0.5	ns	0.721	0.721	ns	0.333	0.5	ns
Valine	0.625	0.922	ns	0.492	0.922	ns	0.922	0.922	ns
Glucose	0.922	0.922	ns	0.695	0.922	ns	0.492	0.922	ns
Acetic acid	0.375	0.375	ns	0.053	0.113	ns	0.076	0.113	ns
Formic acid	0.959	0.959	ns	0.359	0.712	ns	0.475	0.712	ns

↑ indicates higher after ‘Broken’ meal, ↓ indicates higher after ’Intact’ meal. P and P.adj indicated the P values before and after adjusting the Benjamini-Hochberg False Discovery Rate (FDR); ns, non-significance; sig., significance.

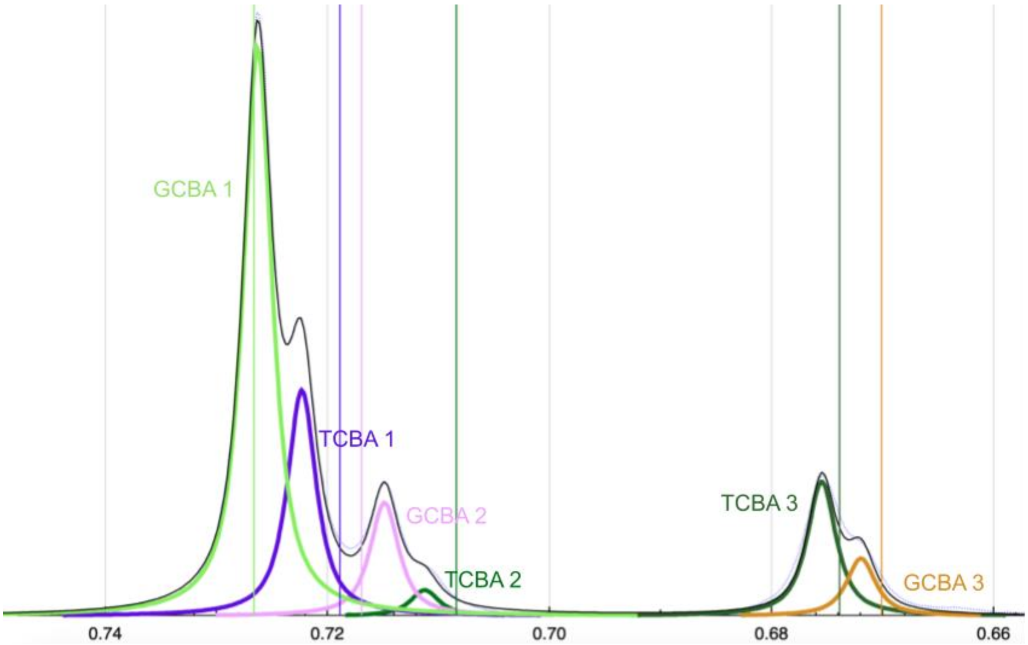

Supplemtary Figure 1. Visualization of algorithmically fitted pseudo-Voigt profiles used for metabolite quantification. The image is a snapshot from our visual assessment tool, displaying a SROI comprising signals from methyl resonances of Bile Acids. The dotted grey line is the experimental spectrum, the black line is the model prediction, and the colored curves are the individual pseudo-Voigt profiles of the signals that constitute the model. The integrals of the colored cuves divided by the number of protons provide the concentrations of the corresponding metabolites.

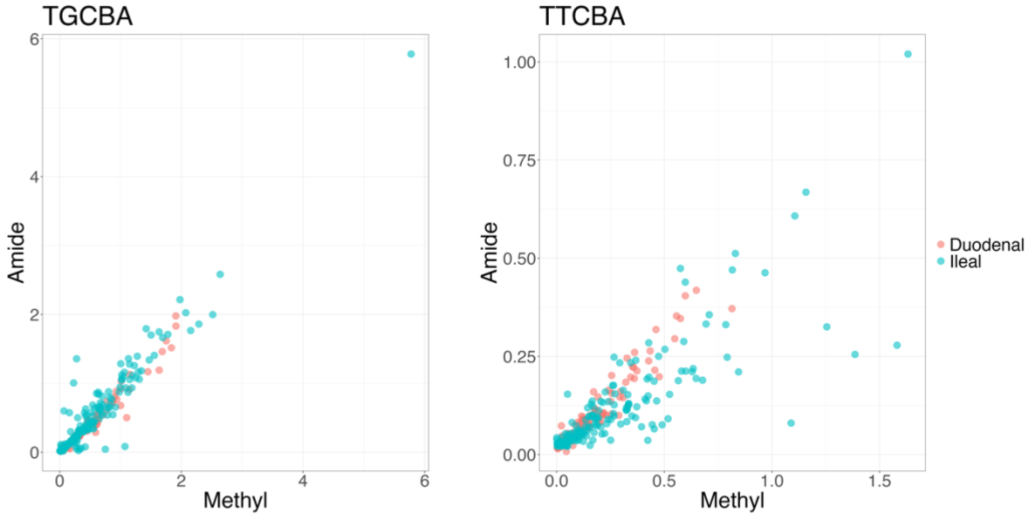

Supplemtary Figure 2. Validation of metabolite quantifications through correlation between separate signals. In this example the horizontal axis represents the aggregate concentrations of Glycine-Conjugated (TGCBA, left) and Taurine-Conjugated (TTCBA, right) Bile Acids computed from the methyl resonance at 0.7 ppm (**Figure S6**), while the vertical axis represents the corresponding values computed from the amide resonance at 7 – 8 ppm. As concentration is independent of the chosen NMR signal, the scatter plots should approach the identity.
