# Supplementary material for: Upper-gastrointestinal tract metabolite profile regulates glycaemic and satiety responses to meals with contrasting structure: a pilot study
Source: Nat Metab. 2025 Jun 20;7(7):1459–75. doi: 10.1038/s42255-025-01309-7 (PMC12286859; doi:10.1038/s42255-025-01309-7)
Supplement: Supplementary file 2 — Reporting Summary [file 42255_2025_1309_MOESM2_ESM.pdf]

nature portfolio

Corresponding author(s): Prof.Gary Frost , Dr.Cathrina Edwards

Last updated by author(s): Apr 11, 2025

Reporting Summary

Nature Portfolio wishes to improve the reproducibility of the work that we publish. This form provides structure for consistency and transparency in reporting. For further information on Nature Portfolio policies, see our Editorial Policies and the Editorial Policy Checklist.

Statistics

For all statistical analyses, confirm that the following items are present in the figure legend, table legend, main text, or Methods section.

n/a	Confirmed	
☐	☒	The exact sample size (n) for each experimental group/condition, given as a discrete number and unit of measurement
☐	☒	A statement on whether measurements were taken from distinct samples or whether the same sample was measured repeatedly
☐	☒	The statistical test(s) used AND whether they are one- or two-sided<br>*Only common tests should be described solely by name; describe more complex techniques in the Methods section.*
☐	☒	A description of all covariates tested
☐	☒	A description of any assumptions or corrections, such as tests of normality and adjustment for multiple comparisons
☐	☒	A full description of the statistical parameters including central tendency (e.g. means) or other basic estimates (e.g. regression coefficient) AND variation (e.g. standard deviation) or associated estimates of uncertainty (e.g. confidence intervals)
☐	☒	For null hypothesis testing, the test statistic (e.g. F, t, r) with confidence intervals, effect sizes, degrees of freedom and P value noted<br>*Give P values as exact values whenever suitable.*
☒	☐	For Bayesian analysis, information on the choice of priors and Markov chain Monte Carlo settings
☒	☐	For hierarchical and complex designs, identification of the appropriate level for tests and full reporting of outcomes
☐	☒	Estimates of effect sizes (e.g. Cohen's d, Pearson's r), indicating how they were calculated

Our web collection on statistics for biologists contains articles on many of the points above.

Software and code

Policy information about availability of computer code

Data collection	no software used for data collection
Data analysis	Software: GraphPad Prism (V9.0, Graphpad Software USA, Biomatters, Ltd), R Studio (V1.4.1106, R Core Team), MATLAB (R2021a, The MathWorks Inc, Natwick, USA), B.I.Quant-PS (Version 2.0.0) and G*Power (Version 3.1.9.6). Algorithm: Statistical Total Correlation Spectroscopy and Subset optimization by reference matching (STOCSY and STORM in MATLAB, https://bitbucket.org/jmp111/storm/src/master/), code for executing the PLS-DA in MATLAB (https://bitbucket.org/jmp111/capls/src/master/) ;Package: 'ComplexHeatmap' and 'plsr' in RStudio

For manuscripts utilizing custom algorithms or software that are central to the research but not yet described in published literature, software must be made available to editors and reviewers. We strongly encourage code deposition in a community repository (e.g. GitHub). See the Nature Portfolio guidelines for submitting code & software for further information.

Data

Policy information about availability of data

All manuscripts must include a data availability statement. This statement should provide the following information, where applicable:

- Accession codes, unique identifiers, or web links for publicly available datasets
- A description of any restrictions on data availability
- For clinical datasets or third party data, please ensure that the statement adheres to our policy

The data reported in this study are available from Mendeley Data Database at https://data.mendeley.com/datasets/4vn35twm9v/1 . Human Metabolome Data Base (HMDB, Human Metabolome Data Base (HMDB; http://hmdb.ca/) and the Biological Magnetic Resonance Data Bank (BMRB; http://www.bmrb.wisc.edu) were used for metabolite identification.

Research involving human participants, their data, or biological material

Policy information about studies with human participants or human data. See also policy information about sex, gender (identity/presentation), and sexual orientation and race, ethnicity and racism.

Reporting on sex and gender	There were 6 male and 4 female adult participants who completed the trial. The gender of participants was determined based on self-report and gender presentation. Due to the small sample size, we did not perform any post hoc sex- and gender based analyses.
Reporting on race, ethnicity, or other socially relevant groupings	No socially relevant categorization was made in the section of study participants.
Population characteristics	We admitted 6 male and 4 female adult participants aged (mean ± SEM) 30.8 y ± 2.4 years with BMI 24.9 ± 0.8 kg/m2 and fasted glucose 4.7 ± 0.1 mmol/L. Participants with an abnormal ECG, screening blood values outside the clinical reference range, a history of cancer, diabetes, gastrointestinal disease and/or requiring medication likely to interfere with metabolic and hormone responses were excluded.
Recruitment	All participants were recruited from the healthy volunteer database of NIHR Imperial Clinical Research Facility. Individuals who expressed interest were invited to complete a pre-screening form and attend an in-person screening visit, during which eligibility was assessed by a medical doctor based on health history, anthropometric measurements, ECGs, and blood test results. This recruitment approach may introduce self-selection bias, as individuals who are more health-conscious or motivated to participate in research may be overrepresented. However, given that this was an acute study conducted in a highly controlled clinical environment, with standardized meals, procedures, and close monitoring, the influence of self-selection bias on the the results is likely to be minimal.
Ethics oversight	This study was approved by the Health Research Authority and London-Camden and King's Cross Research Ethics Committee (REC 19/LO/0962) before the commencement of any study procedures.

Note that full information on the approval of the study protocol must also be provided in the manuscript.

Field-specific reporting

Please select the one below that is the best fit for your research. If you are not sure, read the appropriate sections before making your selection.

☒ Life sciences ☐ Behavioural & social sciences ☐ Ecological, evolutionary & environmental sciences

For a reference copy of the document with all sections, see nature.com/documents/nr-reporting-summary-flat.pdf

Life sciences study design

All studies must disclose on these points even when the disclosure is negative.

Sample size	This was a pilot study. No similar study had been conducted pior to this study so the target of 15 participants was estimated. During the study, the results of Petropoulou, K. et al. 2020 (https://www.nature.com/articles/s43016-020-00159-8) were published. This study, used similar methodology to investigate the impact of resistant starch in upper gastrointestinal digestion with 10 participants, reported significant differences on outcomes such as glycaemia, gut hormone and intestinal starch digestion. Given the interventional nature of the study, and the radiation exposer the volunteers where consenting to, we made the decision to stop the recruitment at 10. Moreover, our study was a randomised crossover study conducted under highly controlled conditions, where participants were in-patients and fully supervised during the entire intervention period. This setup was crucial in ensuring strict adherence to the protocol and enhancing the statistical power.
Data exclusions	No data was excluded from the analysis
Replication	Experiments were conducted in duplicate. All attempts at replication were successful. The coefficients of variation (CV%) for all assays were below 10%.

Randomization	Randomization was performed using a sealed envelope system (Sealed Envelope Ltd. 2022). The randomiszation was performed by an independent researcher who was not involved in this trial.
Blinding	This study followed a double-blinded, randomized crossover design.

Reporting for specific materials, systems and methods

We require information from authors about some types of materials, experimental systems and methods used in many studies. Here, indicate whether each material, system or method listed is relevant to your study. If you are not sure if a list item applies to your research, read the appropriate section before selecting a response.

Materials & experimental systems

n/a	Involved in the study
☐	☒ Antibodies
☒	☐ Eukaryotic cell lines
☒	☐ Palaeontology and archaeology
☒	☐ Animals and other organisms
☐	☒ Clinical data
☒	☐ Dual use research of concern
☒	☐ Plants

Methods

n/a	Involved in the study
☒	☐ ChIP-seq
☒	☐ Flow cytometry
☒	☐ MRI-based neuroimaging

Antibodies

Antibodies used	GLP-1 (106612-94-6 from Bachem); PYY (123583-37-9 from Bachem)
Validation	GLP-1:https://shop.bachem.com/product/4034865/<br>PYY: https://shop.bachem.com/product/4018880/

Clinical data

Policy information about clinical studies

All manuscripts should comply with the ICMJE guidelines for publication of clinical research and a completed CONSORT checklist must be included with all submissions.

Clinical trial registration	Trial registration: ISRCTN18097249
Study protocol	Study protocol has been submitted with the manuscript
Data collection	Location: Imperial College London; Study Start Date: February 2020; Study End Date: April 2021
Outcomes	The primary outcome was the blood gut hormone response. Co-secondary outcomes included intestinal content analysis, blood glucose and insulin response, subjective appetite changes and ab libitum energy intake.<br><br>GIP concentrations in blood were measured using Human GIP ELISA kits (EZHGIP-54K, Merck, UK). GLP-1 and PYY concentrations were assessed using an in-house radio-immunoassay (RIA) method.<br>For intestinal content analysis, gastric and duodenal glucose concentrations were determined using the GLUC-PAP enzymatic kit (Randox Laboratories Ltd., UK), while maltose levels were measured by liquid chromatography–mass spectrometry (LC-MS).<br>Untargeted metabolomic profiling of intestinal contents was conducted using proton nuclear magnetic resonance spectroscopy (1H NMR). Blood glucose was measured using the GLUC-PAP kit, and insulin concentrations were assessed using a Human Insulin-Specific Radioimmunoassay (RIA) kit (HI-14K, Merck, UK). Subjective appetite sensations were evaluated using validated Visual Analogue Scale (VAS) questionnaires administered at predefined time points. Ad libitum energy intake was assessed by offering participants a standardized ad libitum lunch meal, with total intake recorded.

Plants

Seed stocks	*Report on the source of all seed stocks or other plant material used. If applicable, state the seed stock centre and catalogue number. If plant specimens were collected from the field, describe the collection location, date and sampling procedures.*
Novel plant genotypes	*Describe the methods by which all novel plant genotypes were produced. This includes those generated by transgenic approaches, gene editing, chemical/radiation-based mutagenesis and hybridization. For transgenic lines, describe the transformation method, the number of independent lines analyzed and the generation upon which experiments were performed. For gene-edited lines, describe the editor used, the endogenous sequence targeted for editing, the targeting guide RNA sequence (if applicable) and how the editor was applied.*
Authentication	*Describe any authentication procedures for each seed stock used or novel genotype generated. Describe any experiments used to assess the effect of a mutation and, where applicable, how potential secondary effects (e.g. second site T-DNA insertions, mosiacism, off-target gene editing) were examined.*
