# Supplementary figures and images for: Upper-gastrointestinal tract metabolite profile regulates glycaemic and satiety responses to meals with contrasting structure: a pilot study
Source: Nat Metab. 2025 Jun 20;7(7):1459–75. doi: 10.1038/s42255-025-01309-7 (PMC12286859; doi:10.1038/s42255-025-01309-7)

A

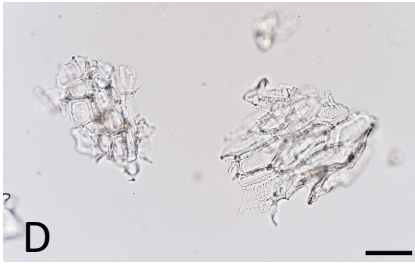

B

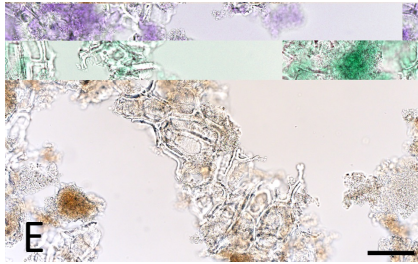

C

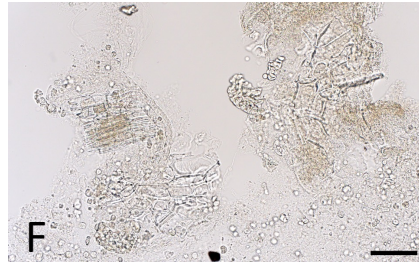

D

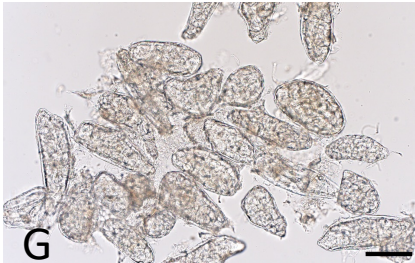

E

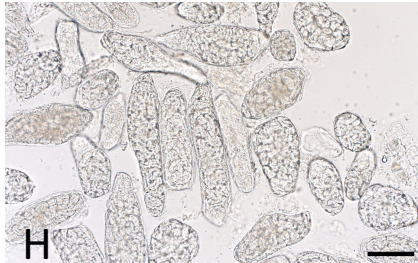

F

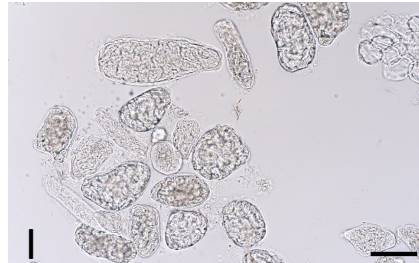

G

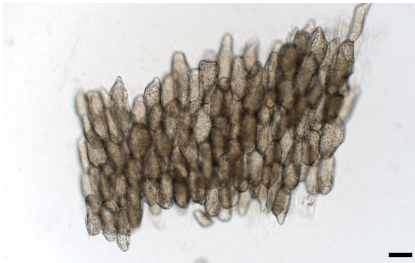

H

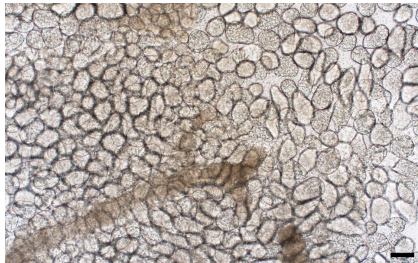

I

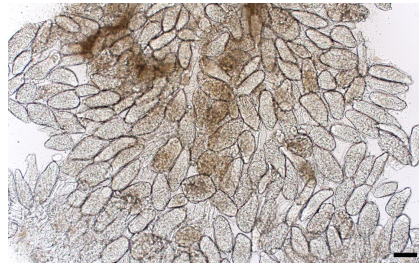

Supplement: Supplementary file 6 — Unprocessed microscopic images. [file 42255_2025_1309_MOESM6_ESM.pdf]
